# Comparing the Representation of a Simple Visual Stimulus across the Cerebellar Network

**DOI:** 10.1523/ENEURO.0023-24.2024

**Published:** 2024-07-16

**Authors:** Ot Prat, Luigi Petrucco, Vilim Štih, Ruben Portugues

**Affiliations:** ^1^Max Planck Institute of Neurobiology, Sensorimotor Control Research Group, Martinsried 82152, Germany; ^2^Institute of Neuroscience, Technical University of Munich, Munich 80805, Germany; ^3^Istituto Italiano di Tecnologia, Center for Neuroscience and Cognitive Systems, Rovereto 38068, Italy; ^4^EnliteAI GmbH, Wien 1010, Austria; ^5^Munich Cluster for Systems Neurology (SyNergy), Munich 81377, Germany

**Keywords:** cerebellum, imaging, larval zebrafish, luminance, temporal patterning

## Abstract

The cerebellum is a conserved structure of the vertebrate brain involved in the timing and calibration of movements. Its function is supported by the convergence of fibers from granule cells (GCs) and inferior olive neurons (IONs) onto Purkinje cells (PCs). Theories of cerebellar function postulate that IONs convey error signals to PCs that, paired with the contextual information provided by GCs, can instruct motor learning. Here, we use the larval zebrafish to investigate (1) how sensory representations of the same stimulus vary across GCs and IONs and (2) how PC activity reflects these two different input streams. We use population calcium imaging to measure ION and GC responses to flashes of diverse luminance and duration. First, we observe that GCs show tonic and graded responses, as opposed to IONs, whose activity peaks mostly at luminance transitions, consistently with the notion that GCs and IONs encode context and error information, respectively. Second, we show that GC activity is patterned over time: some neurons exhibit sustained responses for the entire duration of the stimulus, while in others activity ramps up with slow time constants. This activity could provide a substrate for time representation in the cerebellum. Together, our observations give support to the notion of an error signal coming from IONs and provide the first experimental evidence for a temporal patterning of GC activity over many seconds.

## Significance Statement

The cerebellum is an important brain structure shared by all vertebrates, playing a crucial role in sensorimotor behavior. By using calcium imaging in larval zebrafish, we investigated sensory responses across granule cells (GCs), Purkinje cells, and inferior olive neurons (IONs). We describe how GCs show tonic and graded responses, as opposed to IONs, more active at stimulus transition times, confirming the notion that GCs and IONs encode context and error information, respectively. We also show how time could be represented in the cerebellum by patterned activity in GCs. Together, our observations support the notion of an error signal coming from IONs and provide the first experimental evidence for a temporal patterning of GC activity over many seconds.

## Introduction

To orchestrate appropriate motor reactions and maximize the survival chances of the organism, brains need to generate sensory representations of the environment and its changes. The cerebellum has long been considered one of the main regions involved in the integration of sensory and motor representations and plays a central role in motor coordination, the control of fine motor skills, and the calibration of reflexes and motor learning (Ito, 2006). These abilities are supported by the convergence of two information streams onto Purkinje cells (PCs): parallel fibers and climbing fibers (Eccles et al., 1967).

Parallel fibers run in the molecular layer of the cerebellum and originate from granule cells (GCs), the most abundant cell type in the human brain (Williams and Herrup, 1988). Thousands of parallel fibers establish excitatory synapses onto a single PC. Inspired by these numbers, the first theories of cerebellar function proposed that GCs sparsely code and expand the dimensionality of inputs, which originate in precerebellar nuclei and arrive at GCs via mossy fibers (Marr, 1969; Albus, 1971). This high-dimensional representation of sensory information allows selective and differential weighting of stimulus properties and the acquisition of context-dependent cerebellar-guided behavioral responses (Dean et al., 2010). The wide scope of modalities that can be represented by GCs encompasses sensorimotor (Knogler et al., 2017), somatosensory (Arenz et al., 2009), and even predictive (Giovannucci et al., 2017; Wagner et al., 2017) information, but recent studies have challenged the notion of sparse representations (Giovannucci et al., 2017; Knogler et al., 2017; Wagner et al., 2017). In addition to sparse coding, theories postulate that GCs can provide temporal information to PCs, as feedforward control of movement depends on the ability of the cerebellum to perform temporally specific learning (Kawato and Gomi, 1992; Ohyama et al., 2003) although see Johansson et al. (2014). The large number of GCs makes them suited to encode this information, but while temporal patterning of GC responses has been assumed in multiple theoretical studies (Buonomano and Mauk, 1994; Medina et al., 2000; Medina and Mauk, 2000), little empirical evidence has been found so far (but see Kennedy et al., 2014).

The second stream arriving at PCs via climbing fibers originates in inferior olive neurons (IONs). Each PC is initially contacted by a few climbing fibers and then pruned during development to a single one (Crepel, 1982; Bosman and Konnerth, 2009; Hsieh et al., 2014), which fires at low frequency and evokes large and sustained depolarizations (Eccles et al., 1966). Most widespread theories argue that climbing fibers convey error signals (D’Angelo et al., 2011; Ito, 2013). These, paired with the contextual information provided by parallel fibers, can guide motor learning (Marr, 1969; Albus, 1971) through long term depression at the parallel fiber to the Purkinje cell synapse (Ito, 2000).

To characterize and compare the functional properties of these convergent information streams, we studied them in the olivocerebellar circuit of the zebrafish larvae. The zebrafish cerebellum shares the same basic circuitry with the mammalian cerebellar cortex (Bae et al., 2009; Hamling and Tobias, 2015) but contains a greatly reduced number of neurons, and its cells are already differentiated by 5 d postfertilization (dpf; Hibi and Shimizu, 2012). Moreover, the fish cerebellum has been implicated in several sensory–motor behaviors such as the optokinetic reflex (Portugues et al., 2014) and optomotor response adaptation (Ahrens et al., 2012; Markov et al., 2021).

Taking advantage of the optical transparency of the fish larvae and the availability of transgenic lines that express in specific cell types of the circuit, here we sequentially monitored whole-field luminance responses of all main cerebellar subpopulations in awake and sensing zebrafish larvae. These experiments allowed us to study how GCs and IONs convey different information about the same stimulus. We observed that while GCs showed sustained responses carrying accurate information about the current state of the sensory input, IONs responded mostly to stimulus changes. Moreover, we were able to observe the signs of temporal integration in GC responses, suggesting that their activity could provide a basis for temporal coding in the cerebellum. Finally, we investigated how GC and ION response profiles are integrated at the level of PCs.

## Materials and Methods

### Zebrafish lines

The following experiments were performed with 6–8 dpf zebrafish (*Danio rerio*) larvae. Before experimentation, larvae were kept on a 14 h light/10 h dark cycle and at a constant temperature of 28°C. Three types of transgenic lines in a nacre (*mitf*−/−; Lister et al., 1999) genetic background were used for functional imaging experiments. For GC and ION imaging, a modified fast calcium indicator under the UAS promoter UAS:GCaMP6fEF05 (Felix et al., 2021) was expressed either under a GC-specific Tg(gSA2AzGFF152B) (Takeuchi et al., 2015) or ION-specific Tg(hspGFFDMC28C) (Takeuchi et al., 2015) Gal4 promoter. For PC imaging, GCaMP6s was expressed under a direct PC promoter Tg(PC:GCaMP6f) (Knogler et al., 2019). For behavioral experiments, the Tuepfel long-fin wild-type strain was used. Experiments were performed at a developmental stage at which no sexual differences are yet observable. All procedures related to animal handling were conducted following protocols approved by the Technische Universität München and the Regierung von Oberbayern.

### Stimuli

All stimuli were presented to the lower part of the visual field of the fish, to cover the whole base of the dish where the larvae were embedded or swimming. These stimuli consisted of different combinations of sharp luminance changes, and their display was controlled with Stytra (Štih et al., 2019). For the experiments presented in this study, two different protocols were used. The “steps” protocol consisted of 5 s luminance steps of three different brightness levels (corresponding to 5, 20, and 100% of the maximal luminance, chosen to map quasi-logarithmically the dynamic range of the projector) with 7 s interstimulus intervals. All the possible luminance transitions between the different levels were sampled in each repetition. The “flashes” protocol consisted of 3, 7, and 21 s flashes of maximal brightness, interspaced by 7 s interstimulus intervals. The projectors were calibrated before the design of the stimuli: the lowest and highest luminances (corresponding to 0 and 255 pixel brightness values) were 0.3 and 55.7 lux, respectively.

### Freely swimming behavioral experiments

For behavioral experiments, 6–7 dpf fish larvae were placed three at a time in ∼1 cm of water in a rectangular-shaped arena cut in a 1% agarose matrix in 88 mm Petri dishes. The dish was placed on top of a light-diffusing screen mounted on a clear acrylic support, illuminated from below using an array of infrared LEDs (setup fully described by Štih et al. (2019) and in Stytra documentation). Larvae were tracked online at 500 Hz using a high-speed camera (Ximea MQ013MG-ON) with Stytra. Visual stimulation was displayed from below using an Asus P2E microprojector and a cold mirror (Edmund Optics). A red long-pass filter (Kodak Wratten No. 25) was placed after the projector to match the absolute luminance values and wavelength of the imaging experiment stimulation. In freely swimming experiments, we used the “steps” protocol, presented a total of 36 times (1 h total duration).

### Functional imaging experiments

For functional imaging, larvae were placed in 35 mm Petri dishes and embedded in 2.5% agarose. The dishes were placed onto an acrylic support, on top of a light-diffusing screen, and either the cerebellum or the inferior olive was systematically imaged with a custom-built two-photon microscope. For excitation, a Ti:sapphire laser (Spectra-Physics Mai Tai) tuned to a 905 nm wavelength was used. Visual stimuli were projected from below at a rate of 60 frames per second using an Asus P2E microprojector, and a red long-pass filter (Kodak Wratten No. 25) was placed in front of the projector. Imaging frames were acquired every 248.88 ms (for cerebellum imaging) or 246.38 ms (for inferior olive imaging). The protocol was presented six times while acquiring every plane, and then the focus was shifted ventrally to 1.5 μm (for cerebellum imaging) or 1 μm (for inferior olive imaging), and the process was repeated. For every fish, ∼50–70 planes were imaged.

### Image processing

#### Alignment

2P data were first aligned with a plane-wise rigid transformation. A reference image for each plane was computed as the average of 50 frames, and displacement of each plane from the reference was found through image cross-correlation and corrected with rigid translation. Frames with a correlation peak of >10 microns from the center, usually coming from motion artifacts, were set to NaN and discarded in the following analyses. To correct for shifts happening between planes, a similar procedure was followed calculating shifts from one plane to the next using cross-correlation between consecutive plane averages.

#### ROI extraction

For ROI extraction, we used a combination of approaches. For GCs and PCs, as the number of cells is high, manual segmentation would be unfeasible; but, as responsive cells were generally reliable, it was possible to reconstruct individual ROIs spanning multiple planes, thanks to the similarity of the signal between planes. For IONs, the sparse activity made it difficult to match ROI activity across planes based on correlation, but the low number of cells (∼80 per fish) made manual segmentation feasible.

For automatic ROI extraction, we used the same iterative procedure described by Markov et al. (2021). Briefly, a spatial correlation map was computed where each pixel was assigned a value corresponding to the correlations over time between its fluorescence and the average fluorescence of the eight neighbor pixels. Then, starting from the yet unassigned pixel with the highest correlation value (seed), neighbor pixels were included in the ROIs if their fluorescence correlation with the average fluorescence of the ROI grown so far passed a certain threshold.

For manual ROI extraction, a custom Python graphical user interface was used to draw ROIs on the correlation maps.

### Response analysis

All the analysis on the obtained traces was done in Python; Jupyter Notebooks generating all the figures of the paper are available in the linked repository.

#### Reliability index and filtering

To find a measure of responsiveness to the presented stimulus as independent as possible from the specific response profile, we calculated for each ROI the average correlation of the responses across all individual presentations of the stimulus. The distribution of the obtained correlations had a peak close to zero and a second peak (or a long tail) of positive correlations corresponding to responsive cells. To use an objective criterion to select responsive cells, we used Otsu's method from the SciPy package to set a threshold on the obtained histogram.

#### Hierarchical clustering

Next, we calculated the mean response for each responsive ROI, and we used the ward algorithm for hierarchical clustering (from the SciPy package) to cluster them. We manually specify a cut on the clustering tree to obtain the discrete clusters that we use in the rest of the analysis. We arbitrarily decided on a threshold that was low enough to include all clusters with qualitatively different changes in their response properties.

#### Regressor analysis

For regressor-based analysis, we manually designed a set of regressors starting from either luminance profiles with a gamma correction of 1, ½, or 2; the difference between the luminance profile with Gamma 2 and with Gamma 1 (imitating intermediate luminance responses); and ON, OFF, and combined ON and OFF transitions (Extended Data Fig. 3-1). The obtained regressors were then convolved with a kernel of 3 s, to match the temporal response of GCaMP6fEF05. For each cell, Pearson’s correlation with all regressors was computed.

#### Center of mass sorting

The center of mass (COM) was defined as the point in time where the integral of the neuronal trace reached half of its total integral value. To cross-validate the plots, the value for each ROI was estimated based on the half of neuronal responses that were not used for the plotting (Extended Data Fig. 5-1*A*).

#### Decoding

To decode different information from the cell activities, we used standard methods from the scikit-learn Python package (Pedregosa et al., 2011). Every decoding analysis was trained on 10 randomly chosen trials and tested on 2 others (only cells with data from 12 or more trials were used in the analysis). Ridge regression was used to decode the time since stimulus onset (Fig. 5*E*). Kernel-based support vector regression was used as shown in Figure 3*D*. Grid search over the one regularization parameter was performed in all cases by leaving out each of the 10 training trials. For all decoding analyses, we have to caution that the population activity used to decode the populations is constructed. Neurons were sampled from different repetitions and animals, therefore destroying correlations between neurons. In many cases, such correlations, often termed noise correlations (because these remain unaccounted for after taking out stimulus responses) can have a significant impact on the decoding quality (Moreno-Bote et al., 2014). The decoding analysis is provided together with the rest of the analysis code.

10.1523/ENEURO.0023-24.2024.d1 Extended Data Download Extended Data, ZIP file.

#### Model fitting

For the PC modeling analysis, we used the data acquired with the “steps” protocol. First, we calculated average responses from all the GC and ION clusters (eight clusters of GCs and six clusters of IONs). To address potential differences in time constants between calcium sensors, we deconvolved average responses using the GCaMP6fEF05 kernel and convolved using the GCaMP6s kernel to match the sensor used in PCs. The resulting average traces were normalized to be strictly positive and with integral 1. Then, the traces from luminance responsive PCs were *Z*-scored on a trial-by-trial base, high-pass filtered with a very low cutoff frequency (1/80 Hz) to remove slow drifts, and smoothed with a 3 pts mean boxcar window to reduce noise.

The function that was optimized for each cell was the following:

$$PC_{i}(t)={\mathrm{offset}}_{i}+\mathrm{GCcoeff}s_{\mathrm{ij}}.\mathrm{GCregressor}s_{j}\;(t)+\mathrm{IONcoeff}s_{\mathrm{ik}}.\mathrm{IONregressor}s_{k}(t)\;\;$$

where:

-
$PC_{i}(t)$ is the trace for the *i*th PC cell.
-
${\mathrm{GCregressor}}_{\mathrm{sj}}(t)$ and ${\mathrm{IONregressor}}_{\mathrm{sk}}(t)$ are the GC and ION regressors respectively.

The optimization was done on the following parameters:

-
offset: a constant term, bound to be between −5 and 5
-
coeffsGC: coefficients for GC regressors, bound to be between 0 and 1,000
-
coeffsION: coefficients for ION regressors, bound to be between 0 and 1,000

The large coefficient boundaries come from the different normalizations applied on regressors—norm—and on trace *Z*-scoring.

For each cell, two stimulus repetitions (“test” data) were left out to be the final test traces for the analysis and excluded from the entire fitting process. The remaining repetitions (four or more, for cells spanning multiple planes) were used to find the regularization term and do the fit.

To find the L1 regularization lambda parameter, we used leave-one-out cross-validation to calculate fit costs over a logarithmic sweep of lambdas between 10^−7^ and 10^−2^. We trained the model on all the fit traces but one, and we calculated the fit cost on the remaining trace and took the average value across all the left-out traces. Then, we calculated the average lambda and used it for all cells, to allow for a more appropriate comparison of the fit parameters across cells.

After the cross-validation of regularization lambda, the parameters were fit over all the “fit” repetitions. The “test” repetitions were then used to estimate the final fit performance for all the plots in Extended Data Figure 6-2.

To quantify the relative contribution of GC and ION clusters across the PC population, we computed for each cell a GC/ION ratio index going from −1 (only ION clusters with nonzero weights) to +1 (only GC clusters with nonzero weights).

### Behavioral analysis

To analyze the behavioral responses to the luminance transitions, we started with raw velocity traces saved by Stytra. As zebrafish larvae swim in discrete events called bouts, the fish speed was thresholded to extract individual bout events. The time occurrence of each bout event relative to the presented stimulus was binned, and a histogram of bout times was obtained for each fish (Extended Data Fig. 1-1*A*). To assess whether the decrease of bout probability after each luminance OFF transition was statistically significant, we bootstrapped a distribution of bout probabilities from the histogram of each fish and compared the average bout probability 1.5 s after the OFF transition with the first percentile of the bootstrapped distribution (Extended Data Fig. 1-1*C*). In all but three fish, the post-transition probability was lower than the first percentile of the histogram (Extended Data Fig. 1-1*C,D*).

### Data and code availability

All data and code to reproduce the manuscript has been made public:

- All the source data can be found at https://zenodo.org/records/7071735.
- Analysis code including code that generates all the figures in the paper can be found at https://zenodo.org/records/11208720 and is available as Extended data.

## Results

### Experiment description and anatomy

**Figure 1.**
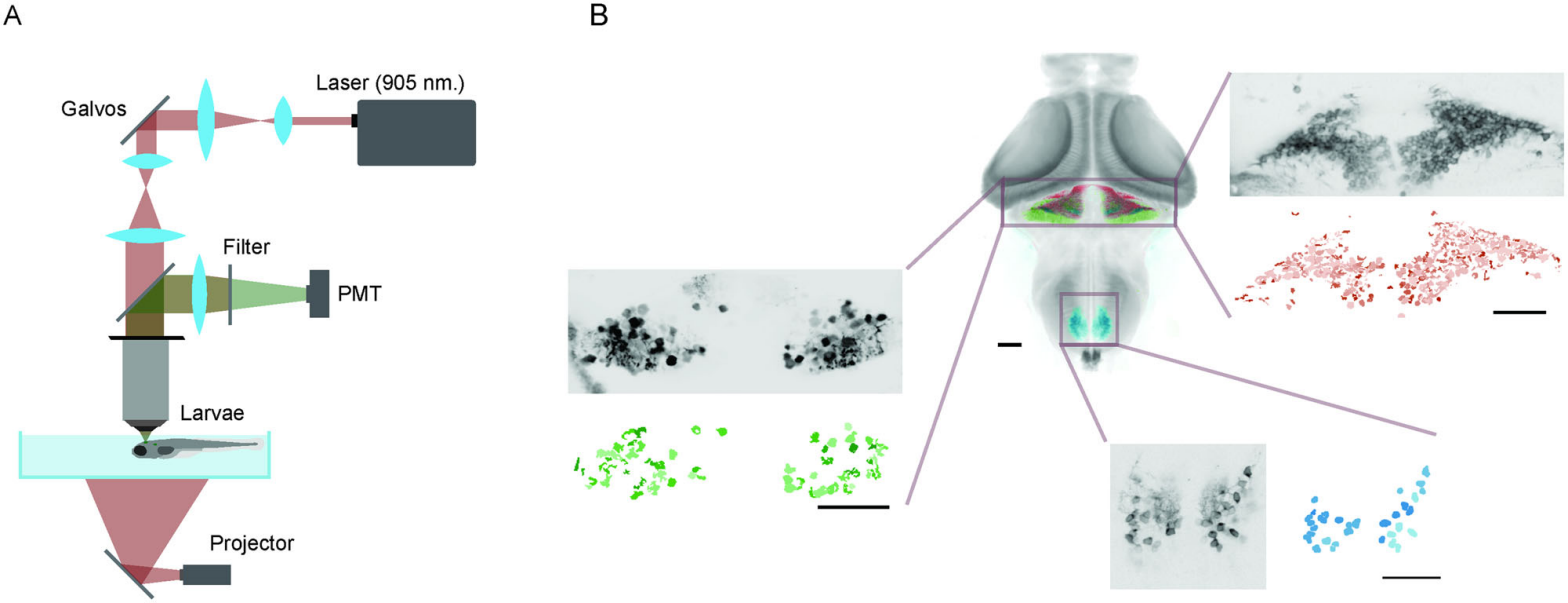
Experiment description and anatomy. ***A***, Experimental setup: a two-photon microscope was used to image 7 dpf head-restrained larvae while flashes of different luminance were projected on a screen below. ***B***, Composite image showing stacks from GCs (red), IONs (in blue), and PCs (green) registered on a reference anatomy with the whole brain of a 7 dpf larva (in gray). In the enlargements, examples of the raw anatomy (in gray) and the segmented ROIs from a two-photon imaging session (in red, blue, and green for GCs, IONs, and PCs, respectively). See Extended Data Figure 1-1 for more details on the behavior elicited.

We exploited the optical transparency and the genetic amenability of the larval zebrafish to follow how information over a simple visual stimulus is processed and transformed through different elements of the cerebellar circuitry. We employed two-photon microscopy in restrained zebrafish larvae to monitor neuronal activity while presenting them with visual stimuli projected on a screen below (Fig. 1*A*). Larvae expressed GCaMP6s or a modified slow version of GCaMP6f (GCaMP6fEF05) in either GCs, PCs, or IONs (see Materials and Methods). The entire volume of either the cerebellum or the inferior olive was scanned in 1 μm (for the inferior olive) or 1.5 μm (for the cerebellum)—spaced planes. In this way, we were able to acquire the responses of a large fraction of the GC, PC, or ION populations (Fig. 1*B*).

10.1523/ENEURO.0023-24.2024.f1-1 Figure 1-1 A) Average normalized bout probability for each individual fish across all stimulus presentations (heatmap), and average probability across all fish (blue line). B) Bout probability changes on a 3.5  s window around luminance offsets. Average bout probability across all off transitions is shown for each fish in gray lines, and the average bout probability across all fish is plotted in blue. C) The average bout probability at offset (during the 1.5  s following luminance changes, green line) across all fish, compared to the 1st percentile (red line) of a dataset generated via bootstrapping. D) Difference between the bout probability at offset and the 1st percentile for the bootstrapping analysis performed individually on each fish. Fish labeled in green correspond to animals where the bout probability was smaller than the 1st percentile. Download Figure 1-1, TIF file.

Zebrafish larvae are highly visual animals, and previous studies (Knogler et al., 2017) have shown that luminance is very salient for neurons in the fish cerebellum. Therefore, we decided to investigate the diversity of responses across cell types as whole-field luminance was changed over a screen placed below the fish. This protocol elicited only a very mild behavior (Extended Data Fig. 1-1*A*), slightly reducing bout probability after light-to-dark transitions (Extended Data Fig. 1-1*B,D*), allowing us to analyze the sensory representations of the stimulus without the confounding effect of motor-related activity.

### Responses of GCs and IONs to different luminance levels

In the first set of experiments, we investigated the responses of GCs and IONs during a protocol where luminance was changed between four distinct levels. The progression was designed to ensure that transitions between all pairs of luminance levels were sampled (Extended Data Fig. 2-1*A*). The entire sequence was presented six times during the imaging of every plane, yielding a robust number of repetitions for all ROIs.

#### Granule cells

**Figure 2.**
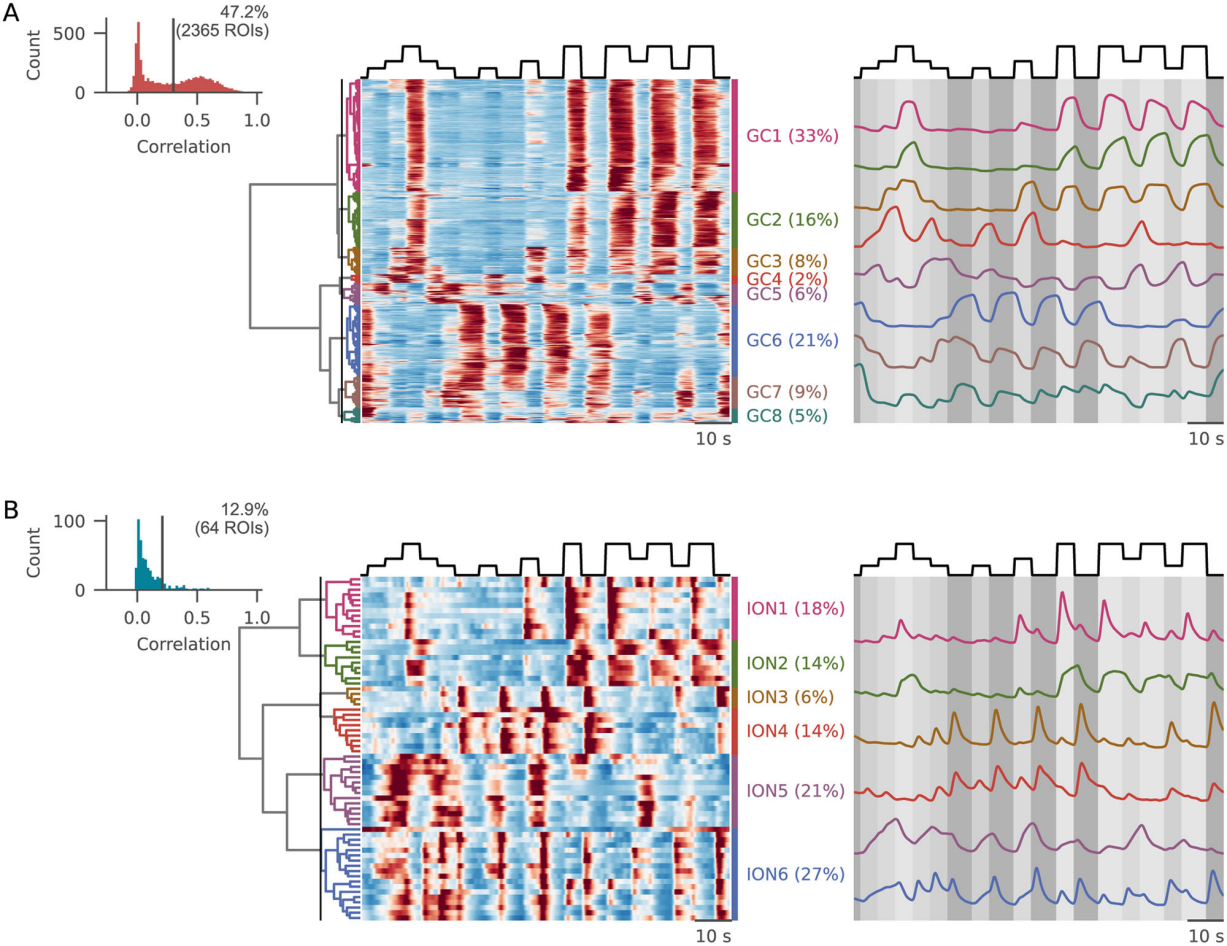
Responses of GCs and IONs to the “luminance steps” protocol. ***A***, Left, Histogram of the average intertrial correlation (average correlation between activity recorded in different trials). The calculated threshold and the relative fraction of active cells are reported on the histogram. Center, Average traces grouped after hierarchical clustering. Cutting the dendrogram at the height marked by the line (on the left) resulted in eight different clusters, whose average activity is shown on the right, overimposed on a shade matching at each timepoint the brightness level displayed. ***B***, Same plot as in ***A***, for IONs. See Extended Data Figure 2-1 for more details.

We collected the responses of 5,013 ROIs from five larvae. To estimate how many cells were reliably engaged by the stimulus, we calculated the average correlation of calcium activity during each pair of stimulus trials (“reliability score”). In the GC population, the distribution of the obtained reliability scores was clearly bimodal (Fig. 2*A*) with a fraction of about half strongly responsive cells (47.2% with an automatic thresholding, see Materials and Methods).

10.1523/ENEURO.0023-24.2024.f2-1 Figure 2-1 A) Schema of the stimulus presented, with the color scheme used for all figures mapped on top of its luminance profile. B) Average fluorescence during the upward luminance steps from minimum luminance (left) and during downward luminance steps from maximum luminance (right) for each GC cluster. C) History dependence of luminance responses for all GC clusters. Average normalized fluorescence during the presentation of the same two intermediate levels of luminance (low intermediate: above, high intermediate: below), compared in epochs when it was reached from a higher (x values) or lower (y values) luminance level. Clusters that deviate from the diagonal are the ones showing the strongest temporal history dependence (color-coded according to Figure 2A). D) Example of GC responses from various clusters, with individual stimulus repetitions (thin lines), average (thick line), and the ROI morphology (on the right). E) Contribution of individual fish to the observed clusters for GCs and IONs. Each color corresponds to one fish. Download Figure 2-1, TIF file.

To explore the diversity of GC responses to the stimulus, we employed hierarchical clustering to classify the average response of all luminance-selective neurons (Fig. 2*A*). The main branching in the distance tree arose from the distinction between ON and OFF cells, hereby defined as the property of an ROI of exhibiting a positive or negative calcium transient in response to an increase in the luminance level. The ON and OFF populations represented 57 and 43% of the responsive ROIs, confirming previous reports (Knogler et al., 2017). However, the presentation of intermediate luminance steps revealed the presence of a minority of ROIs (clusters GC4–GC5) that were specifically recruited by intermediate levels of luminance while silent at either minimum or maximum luminance (Fig. 2*A*, Extended Data Fig. 2-1*B*).

ON and OFF cells did not respond in a homogeneous way to the battery of stimuli. First, we note that granule cell activation was almost always sustained during the entire presentation of each luminance step. Second, cells in both the ON and OFF clusters largely differed in terms of their threshold and saturation points (Extended Data Fig. 2-1*B*). Finally, for some clusters, the activation during the presentation of an intermediate luminance level strongly changed depending on the previous luminance level (Extended Data Fig. 2-1*C*).

#### Inferior olive

We then turned to the analysis of the responses of IONs (495 cells from five fish). IONs were less selective for the luminance stimulus, with a smaller fraction of cells (12.9% with automatic thresholding) exhibiting reliable responses across stimulus presentations. Even if the number of luminance-selective cells was smaller, we could observe a broad diversity of responses in the IONs, with several types of response profiles appearing across fish (Fig. 2*B*). In striking opposition with the sustained responses observed in GCs, ION responses were generally transient, peaking immediately after luminance transitions and quickly decaying afterward. Some IONs were selective to either OFF or ON transitions (clusters ION1 and ION3), while others were recruited by both (ION4). Only a minority of cells showed sustained activation during high luminance periods (ION2, 14%), while two other clusters showed sustained activation during intermediate levels of luminance (ION5 and ION6). Interestingly, in cluster ION6, neurons seemed to combine a sustained activation during intermediate luminance levels with a marked transient activation upon stimulus transitions involving luminance decrements.

Taken together, these observations suggest that the two cerebellar input streams might convey complementary information to PCs. On the one hand, GCs almost always presented sustained activation during the luminance steps, with a broad diversity of responses coming from different thresholds and saturation points. On the other hand, most IONs reacted to sharp transitions in the presented stimulus.

### GCs and IONs respond to stimulus intensity and derivative, respectively

**Figure 3.**
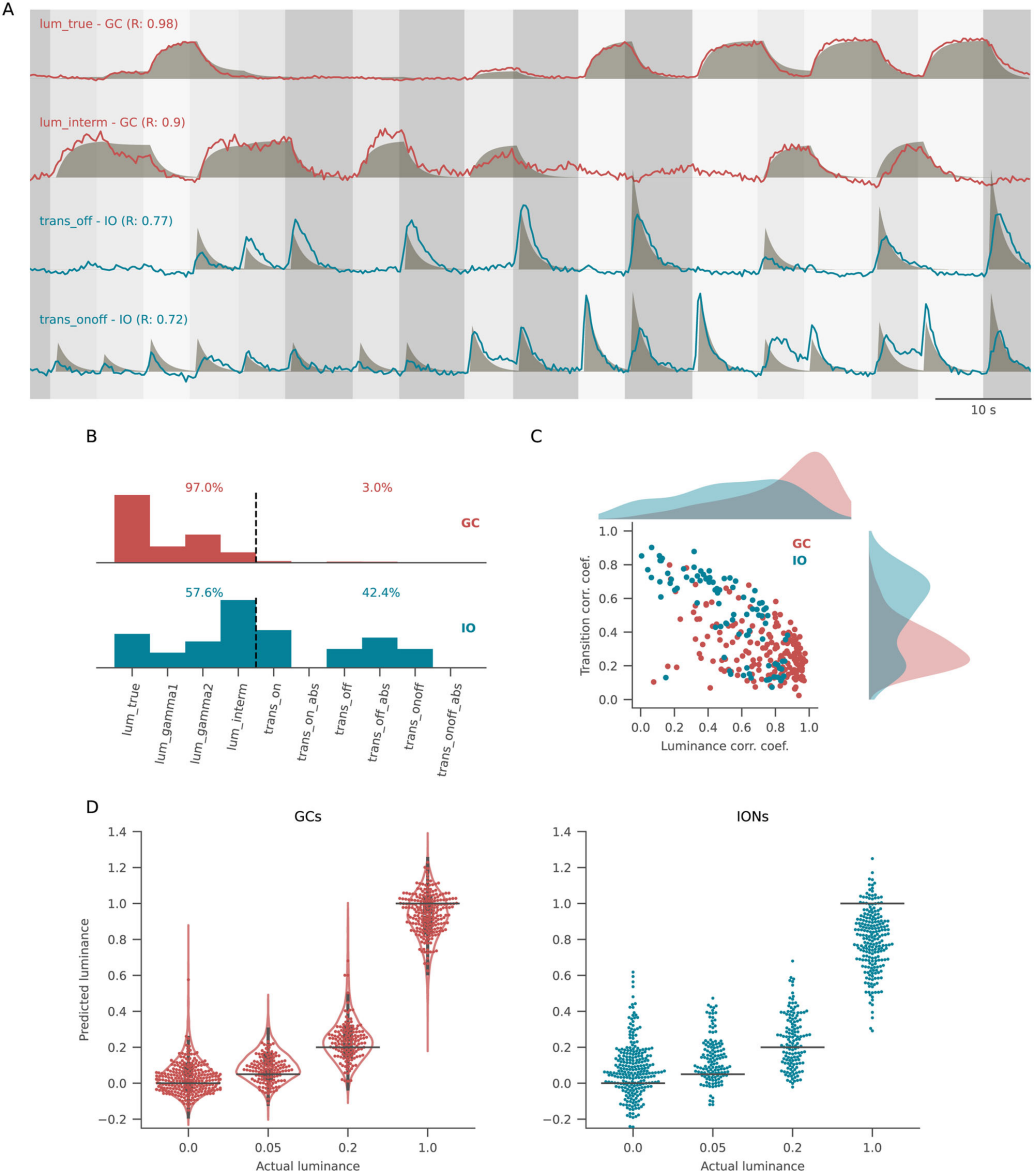
GC and IONs respond to stimulus intensity and derivative. ***A***, Regressor-based analysis was used to calculate the correlation coefficient between each cell’s fluorescence (lines) and a panel of regressors (shades). The plot shows the cells with the highest correlation values for luminance-related (first two rows) and transition-related (second two rows) regressors. ***B***, Histogram of the distribution of best-fitting regressors for GCs (top) and IONs (bottom). Regressors on the left of the dashed line are luminance related, and regressors on the right are transition related. GCs score higher in luminance-related regressors compared with IONs. ***C***, Scatter plot of the best transition-related coefficient and the best luminance-related coefficient for GCs (red) and IONs (blue) and their relative marginal distributions. The GC population has been downsampled randomly to match the number of IONs, while the marginal distributions refer to the entire population. GCs cluster in the bottom right quadrant of the plot (high luminance–regressor correlation, low transition–regressor correlation), while IONs show higher correlation coefficients for transition-related regressors. ***D***, Performance of a nonlinear decoder used to predict luminance values from GCs (left) and IONs (right) activity. Each point in the swarm plot is one frame of the protocol with the corresponding luminance level (horizontal line). For GCs, 20 iterations of the decoding analysis were performed, with the number of GCs downsampled to match that of IONs. The violin plots in the left panel show the average distribution of predictions across all iterations, while the dots correspond to a single representative iteration. See Extended Data Figure 3-1 for more details.

To further investigate this point, we carried out a regression-based analysis of the individual cell responses to better describe these different behaviors. We created a panel of regressors of two different kinds. The first group included regressors obtained by applying different gamma corrections to the raw luminance profile to describe responses with different thresholds and saturation points, plus a regressor obtained by subtracting two luminance traces with different gamma corrections to account for intermediate luminance-selective cells. The second group included regressors created from the luminance derivative: ON transitions, OFF transitions, or both (Fig. 3*A*). We then looked at the distribution of the best predictor for each GC and ION in the dataset. While GCs show large correlations with luminance levels and small correlations with transition-related regressors, IONs were divided into a group showing high luminance-related correlations and a group showing high transition-related correlations. Overall, a larger fraction of ION responses was better predicted by transition-related regressors compared with GC responses (42.4% of IONs vs 3% of GCs, Fig. 3*B,C*). If our observations are true, it should be possible to accurately predict absolute luminance values (the only instantaneous property of the stimulus) from GC activity and more accurately predict transitions of the stimulus from ION activity. By using a nonlinear decoder (a radial-basis function support vector machine regressor), we tried to decode the current luminance level from the activity of IONs or subsets of GCs matching in the size of the ION population. This confirmed that the displayed luminance can be decoded to a higher degree of accuracy from GCs compared with the inferior olive (IO) population, which has more transitory and inconsistent responses (Fig. 3*D*).

10.1523/ENEURO.0023-24.2024.f3-1 Figure 3-1 All regressors used in the regression analysis (shades), and the best scoring ROI for each regressor (lines). While for luminance-related regressors the highest correlation values were always from GCs, for transition-related regressors most of the best scoring ROIs were from IONs. Download Figure 3-1, TIF file.

### Responses of GCs and IONs to flashes of different duration

Looking at the temporal dynamics of the GC responses, we noticed that in some clusters, fluorescence was still markedly increasing at the end of the luminance step (cluster GC2, Fig. 2*A*). We reasoned that this could be the hallmark of temporal integration of the luminance stimulus. Temporal integration has been suggested as a timing mechanism that the cerebellum could exploit to keep track of the period elapsed since stimulus onset, as is required for the acquisition of appropriately timed cerebellar-dependent responses such as eyeblink delay conditioning (Medina and Mauk, 2000). Long time constants in GC responses have been postulated in models to account for timing in the cerebellum (Bullock et al., 1994), but they have never been convincingly observed experimentally.

To unravel temporal integration in the responses of the cerebellar circuitry, we designed a second protocol, consisting of three luminance flashes of different durations (3, 7, and 21 s) at maximal luminance (Extended Data Fig. 4-1*A*). The complete protocol was presented six times in each plane, and the reliability of responses of individual GCs and IONs was assessed as described above.

#### Granule cells

**Figure 4.**
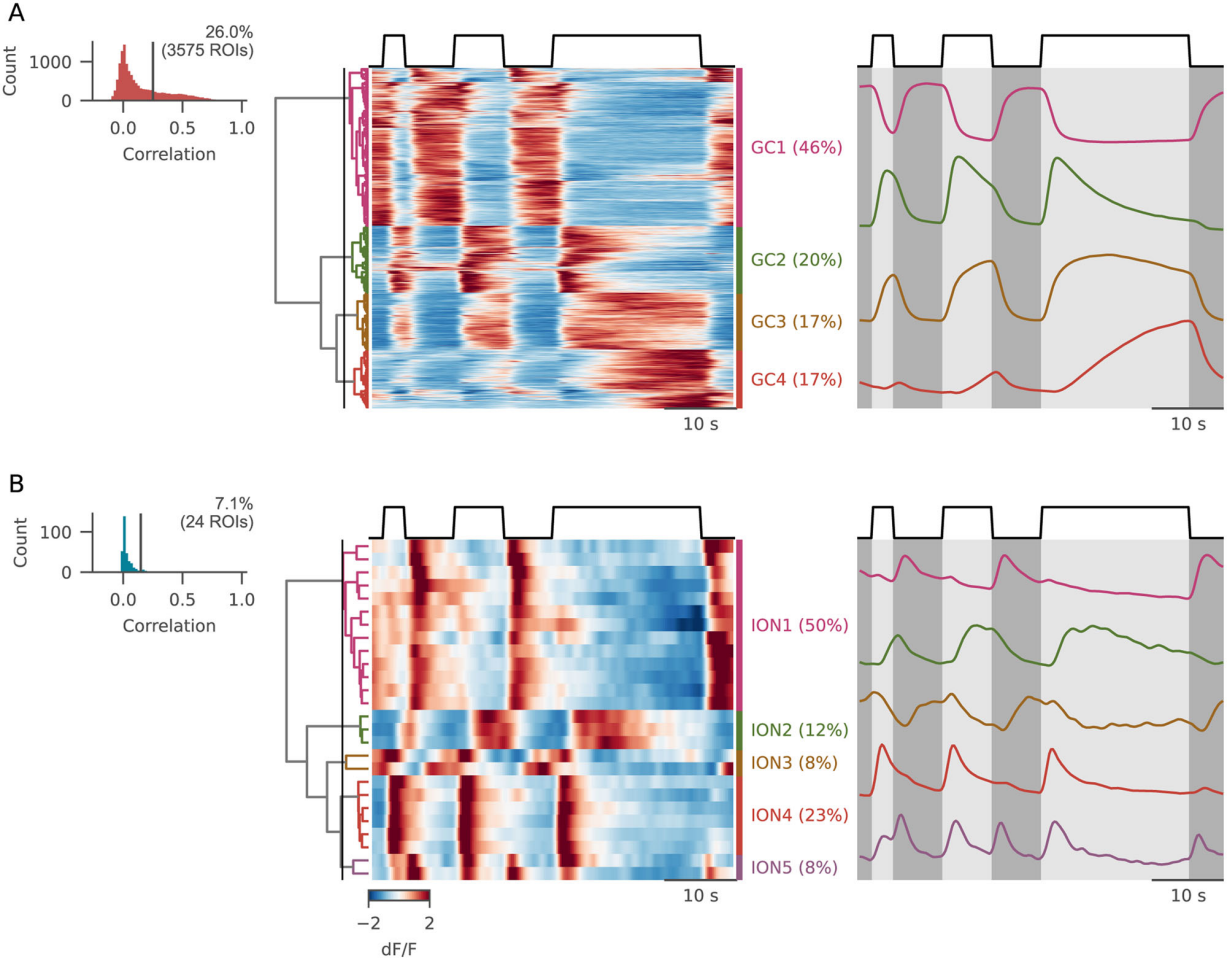
Responses of GCs and IONs to the “flashes” protocol. ***A***, Top-left, Intertrial correlation histogram of GCs for the “flashes” protocol. Center, Cells sorted after hierarchical clustering and (right) average activity for each cluster. ***B***, Same plot as in ***A***, for IONs. See Extended Data Figure 4-1 for more details.

We sampled 13,763 GCs from five fish and selected a fraction of 26% responsive ROIs for successive analyses (Fig. 4*A*). As in the previous protocol, the major split in the GC diversity dendrogram corresponded to the difference between ON and OFF cells, (54 and 46% of the responsive cells, Fig. 4*A*). Interestingly, the population of ON cells contained three clusters whose profiles differed in their temporal dynamics. Cluster GC3 showed the simple sustained activity that was reported in the steps protocol; cluster GC2 rapidly peaked after the flash onset and then decayed to baseline before stimulus end; and cluster GC4 exhibited the response profile expected from a temporal integrator, ramping up slowly for the whole duration of the stimulus presentation. Since the experimental paradigm featured only sustained high luminance periods, we could not compare the kinetics of the luminance ON and the luminance OFF clusters.

10.1523/ENEURO.0023-24.2024.f4-1 Figure 4-1 A) Schema of the stimulus presented, with the color scheme used for all figures mapped on top of its luminance profile. E) Contribution of individual fish to the observed clusters for GCs and IONs. Each color corresponds to one fish. Download Figure 4-1, TIF file.

#### Inferior olive

We imaged 337 IONs from five fish, and, consistent with the previous observations, reliably responsive cells (7.1%) were mostly activated by luminance transitions. Moreover, 50% were selective for negative luminance transitions (cluster ION1), 23% were selective for positive transitions (cluster ION4), and 8% for both transition types (cluster ON5). Only 20% of cells had sustained ON or OFF activations (clusters ION2 and ION3, respectively).

### Granule cell activity is temporally patterned

**Figure 5.**
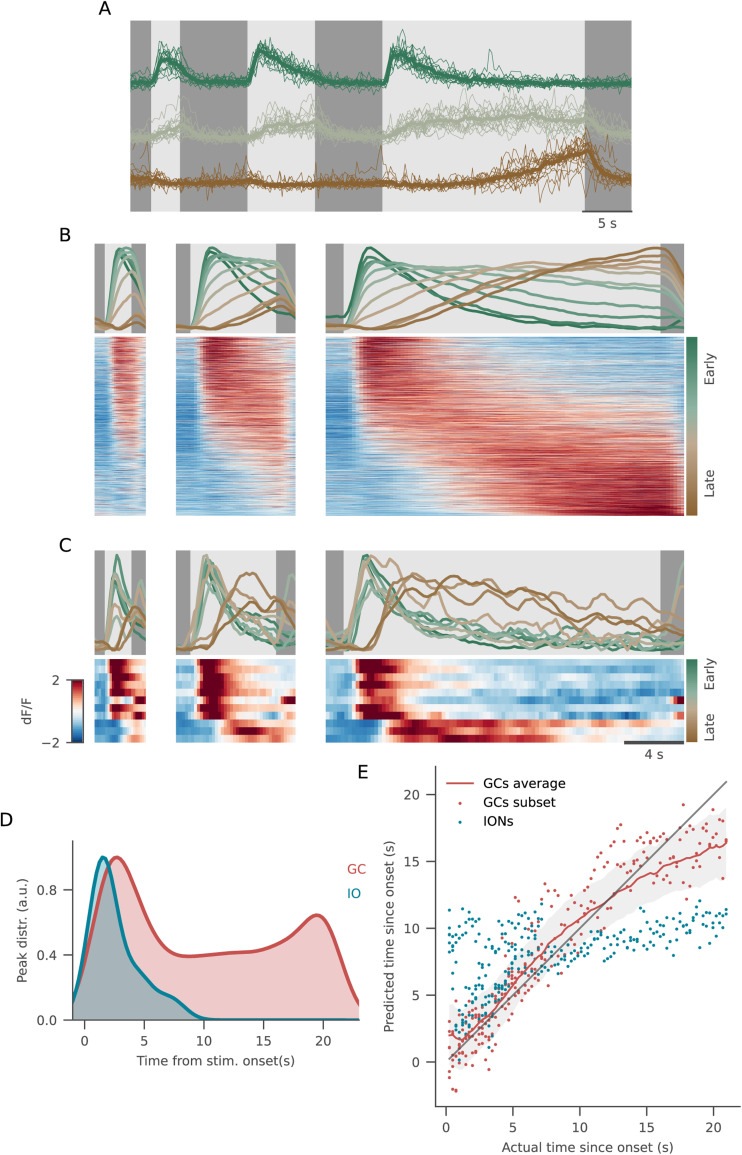
Granule cell activity is temporally patterned. ***A***, Single traces for early- (green), sustained- (gray), and late-responding (brown) ROIs. The average response (thick line) is superimposed on all single repetitions (thin lines) of stimulus presentation. ***B***, GCs included in any of the luminance-excited clusters were then sorted based on their center of mass (COM) during the longest flash, with earlier-responding neurons on the top and late-responding ones on the bottom (bottom panels). The above traces represent the average response for ROIs binned according to the time at which their COM was reached (green for early-responding neurons, brown for late-responding ones). ***C***, Same plot as in ***B***, for IONs. In this case, traces in the top panels correspond to single ROI responses. ***D***, Probability density function describing the time points at which responses of neurons from the two imaged populations reach their maximal response. ***E***, Scatter plot of predicted versus actual time elapsed stimulus onset. Prediction was performed at each frame from ION activity (blue dots) and activity of a random subset of GCs (red dots). The red line and shaded area show average ± standard deviation of predictions from 200 GCs subsets. See Extended Data Figure 5-1 for more details.

Intrigued by the slow temporal dynamics in cluster GC4 (Fig. 4*A*), we decided to further investigate their temporal response properties. Different GCs responded with very different profiles that were consistent across stimulus presentations (Fig. 5*A* and Extended Data Fig. 5-1*C*). Remarkably, some cells from cluster GC4 clearly showed a period of suppressed activity after the stimulus presentation, followed by a positive, “integrator-like” response that could start rising several seconds after the beginning of the stimulus (Fig. 5*A*, third trace). This might reflect the interplay between excitatory and inhibitory drives having different time constants, whose summing effects give rise to the observed diversity in the GC response timing.

10.1523/ENEURO.0023-24.2024.f5-1 Figure 5-1 A) In order to cross-validate our sorting of ROI responses, the COM for each neuron was calculated based on the average response during half of its repetitions, and the other half of the repetitions were used to plot the figures shown in Figure 5. Figure 5-1A shows, for each ROI, the COM calculated on each half of the repetition. B) Histogram of the R2 values between predicted and actual time from stimulus onset, as decoded from 200 subsamples of GCs. The blue line marks the R2 value obtained using the same number of IONs. C) Additional examples for single GC traces exhibiting temporally-patterned responses, from early- to late-responding neurons (color coded from green to brown). Average response (thick lines) is superimposed to single repetitions of stimulus presentation (thin lines). Download Figure 5-1, TIF file.

To look at the distribution of time constants in the responses of GCs from ON clusters (GC2, GC3, and GC4), we sorted them by their cross-validated center of mass (COM) computed on the 21 s flash (see Materials and Methods, Extended Data Fig. 5-1*A*). We observed a continuum between the different response profiles, ranging from early-responding neurons that peak after the flash onset to late-responding neurons with responses slowly ramping up during stimulus presentation (Fig. 5*B*). Most of the neurons reached their maximal activity shortly after stimulus onset, while many others were still ramping up at the time of its offset (Fig. 5*D*). IONs did not show any integrating-like responses, and most ON cells peaked immediately after luminance onsets (Fig. 5*C,D*).

Temporal integration would provide PCs with a representation of time elapsed since stimulus onset, as postulated in models of temporal learning in the cerebellum (Medina and Mauk, 2000; Yamazaki and Tanaka, 2009). Therefore, we examined whether it is possible to linearly decode this time from the activity of an equal number of GCs or IONs. We observed that this decoding is possible to a high degree of accuracy by using the activity of granule cells (Fig. 5*E*), with an *R*^2^ value of ∼0.8. Since the temporal dynamics are nonlinear and share a saturating trend at longer durations, the optimal linear decoder overestimates the time at shorter durations and underestimates longer durations (Fig. 5*E*). The inferior olive cells, on the other hand, show no long-term temporal patterning, and it was therefore not possible to decode the duration of the stimulus presentation (*R*^2^ = 0.29, Extended Data Fig. 5-1*B*).

We conclude that the GCs exhibit diverse temporal dynamics that could provide PCs with a temporal basis for reading out the time since stimulus onset.

### PC responses to luminance stimuli

Both parallel fibers from GCs and climbing fibers from IONs make synaptic contact with PCs. Therefore, we decided to investigate the responses of PCs to the same battery of stimuli, to understand how the afferent cerebellar inputs are integrated when they converge at the level of their postsynaptic target. We used a transgenic line that expresses GCaMP6s selectively in all PCs. It is important to note that the unique biophysics of the PC spiking properties (viz., the difference between complex spikes elicited by climbing fibers and the simple spiking modulated by parallel fibers) might complicate the mapping between intracellular calcium-related fluorescence and spiking activity. Nevertheless, previous data (Knogler et al., 2019) have shown that both complex spikes and bursts of simple spikes induce comparable GCaMP6s fluorescence signals in fish PCs.

**Figure 6.**
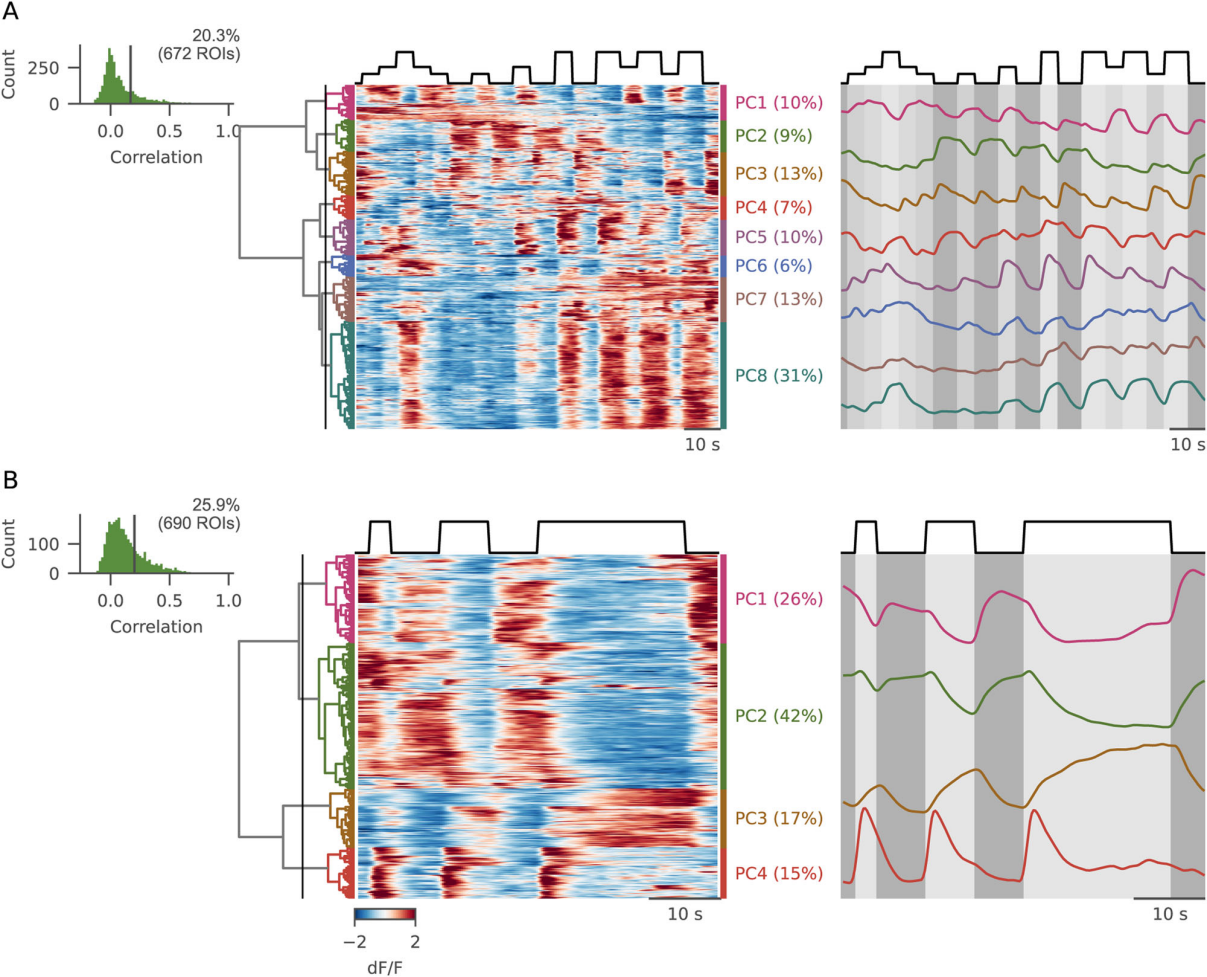
PC responses to luminance stimuli. ***A***, Top-left, Histogram of the average intertrial correlation and its relative threshold. Center, Hierarchical clustering of PC responses to the “steps” protocol, and average activity of the clusters selected after cutting the dendrogram at the height marked by the lines. The shade matches at each timepoint the brightness level displayed. ***B***, Same as in ***A***, for the “flash” protocol. See Extended Data Figures 6-1 and 6-2 for more details.

We first analyzed the responses of PCs to the “steps” protocol (Fig. 6*A*) in 3,318 ROIs from five fish. Of these, 20.3% were recruited by the stimulus. Given the well-characterized nature of synaptic inputs of PCs, we expected to find response profiles reflecting the profiles observed in the GC and the ION populations. PCs showed both ON and OFF selectivity, a fraction of which were selective for intermediate luminance levels, as was observed in GCs (cluster PC1). However, there were significantly more ON cells than OFF cells (67 vs 32%, respectively) suggesting that the former are over-represented in PCs compared with GCs. Importantly, from the clustering approach, we could find only one definite group of cells showing transient, transition-related activity (PC3), while most clusters looked modulated mainly by GC inputs.

10.1523/ENEURO.0023-24.2024.f6-1 Figure 6-1 A) Contribution of individual fish to the observed clusters for PCs in the steps protocol. Each color corresponds to one fish. B) Contribution of individual fish to the observed clusters for PCs in the flashes protocol. Download Figure 6-1, TIF file.

10.1523/ENEURO.0023-24.2024.f6-2 Figure 6-2 A) Schema of the modeling approach. (Left) PCs receive inputs from IONs and GCs. Starting from GC and IO response clusters (center) we linearly combined them trying to reconstruct each PC activity (right). B) Histogram of costs (L^2^) on the test traces from all PCs (green line), compared to a shuffle distribution (brown shade) that was obtained calculating costs after a random regressor-wise reshuffling of the weight matrix (i.e., all values from each individual regressor were kept and reshuffled in new random combinations). A threshold was defined to ensure that only 5% of these random fits could have a lower cost. The green shade indicates the data that were kept after such selection. C) Examples of individual fits. (Left) Average PC response (thick green line) calculated on the individual test repetitions (thin green lines), and reconstructed trace from the model (black line). (Right) Red, trace reconstructed with GC coefficients only; blue: trace reconstructed with IO coefficients only. D) Matrix of weights assigned to each regressor (rows) for all PC ROIs (columns). Vertical gray lines separate PCs clustered together in Figure 6A. E) Correlation between the reliability index and the fit error, including only PCs for which the fit was considered better-than-random. F) Scatter plot showing the relation between the GC/IO weights ratio vs. cell reliability. Each dot represents the two values for a single PC cell, color coded by the GC/IO index. The response of PCs dominated by GC inputs are more reliable compared to cells dominated by IO inputs (Spearman rho: 0.19, p = 5.8*10-4). Download Figure 6-2, TIF file.

Then, we turned to the investigation of PC activity during the “flashes” protocol (Fig. 6*B*), analyzing the responses of 2,267 ROIs from five fish (25.9% responsive). PC activation profiles showed mixed IO and GC features. Just as in GCs, there were many late-responding ROIs that exhibited integrating-like responses (cluster PC3). While the indicator expressed in PC was the slower GCaMP6s, we believe that the difference in the indicator time constant (1–1.5 vs 0.7–1 s) could not account for this difference over long timescales. No cluster showed a constant, sustained response throughout the stimulation period. This is surprising, as the known biophysical properties of PCs would allow them to produce bistable activation (Sengupta and Thirumalai, 2015); it is possible that while stimulus transition could trigger an upstate in some PCs, the transition to downstate could happen before the end of the stimulus. Finally, a large group of ON cells showed only a transient ION-like activation after luminance onset (cluster PC4) that was faster than the one observed in GCs (cluster GC2 in Fig. 4), suggesting input from IONs only.

We finally asked to what extent PC activity could be expressed as a weighted linear sum of the various GC and ION profiles we found. We used multivariate linear regression with cross-validation and LASSO regularization to reconstruct the individual stimulus responses of each PC as a weighted sum of the GC and ION regressors plus an offset term (Extended Data Fig. 6-2*A* and Materials and Methods). As both these populations contact PCs with excitatory synapses, we constrained the model to have only positive weights. The activity of most cells was successfully captured by our simple linear model (Extended Data Fig. 6-2*C*), with ∼57% of cells with a better-than-chance fit cost (Extended Data Fig. 6-2*B*). The distribution of weights across PCs (Extended Data Fig. 6-2*D*) matched the clusters observed in Figure 6*A*, and as expected, there was an inverse relationship between fit cost and cell reliability (Extended Data Fig. 6-2*E*). Overall, we observed that PC calcium responses can be reconstructed as a linear combination of GC and ION response profiles. We investigated this by computing a GC/IO index (see Materials and Methods) quantifying average contributions to individual PC activity (Extended Data Fig. 6-2*F*). Although the calcium response of most PCs was better predicted using loads on GC regressors, the PC population exhibited a broad diversity of GC versus ION loads. Given the differences in the reliability indexes of the GC and the ION responses (Fig. 2*A,B*), we would expect that PCs dominated by GC inputs would show a higher reliability. This was indeed the case, and there was a significant positive correlation between the reliability index of the PC and the GC contribution to its activation (Spearman *ρ* = 0.19, *p* = 0.0006; Extended Data Fig. 6-2*F*). In conclusion, we observed that PC calcium responses can indeed be reconstructed as a linear combination of GC and ION response profiles. Although the activity of most PCs was better predicted using loads on GC regressors, the PC population exhibited a broad diversity of the GC versus ION loads, ranging from ION inputs only to GC inputs only.

## Discussion

Here, we undertook a large-scale investigation of the representations of a simple visual stimulus in the olivocerebellar system of larval zebrafish at the single-cell level. Previous reports have been exploring the responses of individual cell types in the cerebellar circuitry (Knogler et al., 2017, 2019; Harmon et al., 2020; Felix et al., 2021). Building on this previous work, we have systematically explored the responses of IONs and GCs to changes in luminance. Furthermore, we recorded activity in PCs, which receive both GC and ION input, in order to understand how they could combine these signals.

### Stimulus state and transition coding in GCs and IONs

We observed that a large fraction of GCs were recruited by our sensory stimulation, strengthening previous observations that point against the notion of sparse coding in GCs (Giovannucci et al., 2017; Knogler et al., 2017; Wagner et al., 2017). Responses clustered into a few groups which, nevertheless, displayed a diversity of response properties, varying in their sign, threshold, and saturation points. Intriguingly, some GCs were not clearly luminance ON or luminance OFF but were excited by intermediate luminance and inhibited by strong luminance (and vice versa). This activity profile could be easily generated by the interplay between an excitatory input coming via mossy fibers and recurrent inhibition provided by Golgi cells, combined with different threshold and saturation levels in GCs (Bratby et al., 2017). However, we cannot exclude that such responses are not directly inherited by precerebellar inputs (Barker et al., 2017). The diversity observed in these response types would suggest that the representation of the sensory input in the GC layer is not only dense but also allows to reliably decode the presented luminance even from the responses of small subsets of GCs.

On the other side, a smaller fraction of IONs showed consistent responses during our simulation. Importantly, the responses of IONs were much more phasic in nature compared with GC, with a single peak following light-to-dark or dark-to-light transitions (or, less often, both). This observation is in agreement with the idea that climbing fibers report “error” or “salience” signals that can result in cerebellar learning by inducing plasticity on GC to PC synapses, which convey contextual evidence about the state of the environment. As a result, while decoding the presented luminance from ION activity was worse than GC activity–based decoding, ION traces allowed for more reliable discrimination of the stimulus transition times.

Together, these observations confirm the idea that parallel fibers from GCs provide contextual evidence to the cerebellum, while IONs code for sudden changes in the environment, a central tenet of models of learning in the cerebellum.

### Temporal coding in GC responses

The cerebellum is involved in the acquisition and timing of motor sequences. This is exemplified in classic paradigms of delay conditioning, where the conditioned motor response has to be precisely delayed from the onset of the conditioning stimulus. This requires that even the simple conditioning stimuli used in paradigms such as eyeblink conditioning need to evoke GC responses that vary in time so that selective reinforcement of some parallel fiber synapses can modulate the PC firing rate at specific intervals from the onset of the stimulus. Several mechanisms have been proposed to underlie such temporal patterning (Medina and Mauk, 2000; Yamazaki and Tanaka, 2009). On the one hand, in delay line models, the stimulus-elicited activity is delayed by a fixed amount of time by a variable number of synaptic connections in the precerebellar neurons that impinge onto each GC, making the activity of GCs sparse in time, with variable onsets (Moore et al., 1989). On the other hand, in oscillatory models, the responses of GCs are supposed to be oscillatory, with a different characteristic frequency in each GC so that they can sum up to form a basis for arbitrarily delayed PC responses (Gluck et al., 1990). Finally, in spectral models, the temporal patterning arises as a combination of varied membrane time constants in GCs and in inhibitory companion Golgi cells, which generate unique time constants for the ON and OFF phase of each GC (Bullock et al., 1994; Bratby et al., 2017).

Previous studies have reported the existence of temporal patterning over hundreds of milliseconds in the GCs of the electrosensory lobe, a cerebellum-like structure in the brainstem of mormyrid fish (Kennedy et al., 2014). However, this has never been investigated at the whole GC population and at timescales of several seconds. Previous work on larval zebrafish has reported no evidence of temporal patterning in GCs (Knogler et al., 2017), but they analyzed temporal patterning in recordings from a very limited number of neurons. In our experiments, we could find no evidence for temporally sparse or oscillatory activity (in the frequency range observable given the sampling rate, i.e., below 2 Hz). Interestingly, however, we do see a continuum spectrum of delay in the responses, from fast offset GCs to GCs whose activity was suppressed for several seconds before starting to ramp up. This was happening over a much longer timescale than what usually has been investigated in cerebellar studies. We report GCs whose onset was followed by up to 5–6 s the stimulus onset and whose activity was still increasing after 20 s of stimulus presentation. IONs, on the other side, were carrying very little temporal information about the ongoing stimulus. A decoding approach confirmed that accurate prediction of time occurred since stimulus onset was possible from GC activity but not from ION activity. Our data suggest that GCs can indeed provide a base for temporal coding in the cerebellum and strongly support the spectral timing model for temporally specific cerebellar learning.

### PC integration of GC and ION activity

Finally, we aimed to describe the calcium dynamics in PCs as a linear combination of IONs and GCs inputs. It has already been shown that such a linear modeling on afferent inputs can provide a good description of the PC activity (Chen et al., 2017; Tanaka et al., 2019; Markanday et al., 2023). Indeed, we could describe most of the PC calcium activity as a linear weighted sum of responses from IONs and GC clusters. We observed some heterogeneity in the contribution from ION and GC clusters between cells, with some PC cells mostly showing ION-like activity and most cells that were described better by GC regressors, hence suggesting that GC and ION inputs related to the same sensory modality do not necessarily converge over the same PCs. An obvious caveat of our study is the focus on calcium imaging dynamics in PCs, as calcium transients might be produced by different biophysical dynamics for ION-elicited complex spikes and simple spikes. However, it was recently shown that in larval zebrafish, both complex spikes and bursts of simple spikes could be detected using the PC:GCaMP6s line (Knogler et al., 2017, 2019), and we could indeed observe both GC-like and ION-like activity in PCs.

In conclusion, our work provides the first characterization of population-wide responses to the same battery of sensory stimuli in GCs, IONs, and PCs in the cerebellum. This approach gives new insights into how stimulus features and timing are differently represented in the two converging cerebellar pathways and are integrated at the level of PCs and could be leveraged in the future to mechanistically address the involvement of the cerebellum in processing sensorimotor signals.

## Synthesis

Reviewing Editor: David Schoppik, New York University Decisions are customarily a result of the Reviewing Editor and the peer reviewers coming together and discussing their recommendations until a consensus is reached. When revisions are invited, a fact-based synthesis statement explaining their decision and outlining what is needed to prepare a revision will be listed below. The following reviewer(s) agreed to reveal their identity: David Ehrlich, Michael Mauk.

The reviewers felt that the strongest part of the manuscript was the data from granule cells. They argued that if anything, the manuscript underplayed the importance of these findings and encouraged the authors to expand on the characterization. They agreed that the weakest part was the reconstruction of Purkinje cell responses.

Major concerns:

1. The common calcium response in Purkinje cells to simple spike bursts and complex spikes requires more prominence in the interpretation and direct treatment in the Discussion. Complex spikes and simple spikes are almost universally considered independently, and the authors have not presented readers a heuristic for relating the present findings to the literature. At minimum, regarding the claim that PC calcium dynamics can be reconstructed from parallel fiber and climbing fiber inputs, the authors should remind readers in the Discussion that the analysis accounts for the convergence of both input streams on calcium accumulation but is agnostic to the timing of simple and complex spikes. An important comparator will be the paper by Markanday et al Nat Comm 2023. We were not able to find the cited related papers (line 575) as they are omitted from the references list. In this context and considering single innervation, attempting to reconstruct activity of an individual Purkinje cell from a population of inferior olivary neurons is flawed at its premise, and the plausibility and importance of using the entire IO population (versus, per se, a delta function of weights w^IO) would help ground this result in the literature. Relatedly, calling PC responses "activity" in the abstract implies "spiking activity," but one key assumption for relating calcium dynamics to spiking is the presumed direct correlation between intracellular calcium concentration and spike rate. Given the impossibility of disambiguating simple spike bursts and climbing fiber responses, it would be appropriate to assure readers do not interpret these dynamics as spiking activity and therefore to call PC dynamics here "calcium dynamics" or similar.

That said, it is well known a this point that Purkinje cell responses are determined largely by learning and therefore by the relative strengths of the granule cell (and perhaps also inhibitory) synapses. Purkinje cell responses can be made to change dramatically by learning, where the inputs to the neuron do not change, but the Purkinje response changes because of synaptic changes. Given this, we really think there is little value conceptually speaking to this otherwise nicely done analysis. [Ed. note: it's not necessary to remove this analysis, but I recommend considering its framing and presentation relative to these critiques].

2. The claim in the abstract that PC activity can be reconstructed from activity of input cells is misleading because the populations were not recorded in parallel or necessarily in the same animals. There is a discussion of the implications under "decoding" section in the methods (line 200), but this caveat and implications should be included in the Discussion. The distinction is complicated by the statement of "parallel characterization" in line 589; while the responses were parallel in the sense that they were all to the same stimulus array, it would be easy to interpret this claim to mean parallel in time. The sequential nature of the recordings does not diminish the findings here but the authors should be acknowledge this feature of the data collection outside of the Methods.

4. Presentation of the temporal properties would be improved by avoiding the use of smoothing functions. Those smoothed traces give the appearance of gradual increases in activity, even for granule cells that fire at the very onsets of the visual stimuli. Because the consistency of the responses seems strong, presenting these as unsmoothed peri-stimulus time histograms would give a more accurate portrayal of the responding.

Minor concerns:

1. To support the important claim that there was "no consistent anatomical organization in the localization" of granule cell responses (line 308), it would be useful to see granule cell responses organized by subregion in the extended data.

2. It would be helpful for interpreting temporal differences between cell type responses (e.g. line 450) if the authors included specifics on calcium indicator kinetics for the two indicators used. Relatedly, it would be relevant to discuss the lack of sustained responses in PCs in light of their bistability (Sengupta and Thirumalai, 2015).

3. The final sentence of the abstract regarding temporal patterning distorts the novelty of the finding simply because the population in the electrosensory lobe in Kennedy et al are also GCs. The authors should find other wording to highlight the significance of discovering a temporal basis set in the cerebellum proper. Similarly, the description of the Kennedy paper as showing "hints of temporal coding" in granule cells is needlessly vague - theirs was strong evidence for temporal coding in a related but distinct population of cells.

4. The point in line 33 that "efficient sensory representations" are required for motor responses seems overstated and should be supported with a citation.

5. Line 40 reads as though granule cells are situated in the molecular layer

6. Given the developing circuit under consideration in the study and importance of IO-PC connectivity for the interpretation of the PC integration analysis, it is worth mentioning the exception of multi-innervation during development on line 62.

7. The claim that zebrafish cerebellar neurons are mature by 5 dpf (line 72) is confusing as they are still growing. Perhaps the authors mean "differentiated"?

8. The claim of "no evidence for... oscillatory activity" deserves a caveat that identifying oscillations above 2Hz are precluded by the sampling rate.

9. Figure 2 title references PCs but they are not included here.

Line 145 "where" typo

Line 146 "and" typo

Line 373 typo "corresponded mapped"

Line 26 "ION" typo

## Author Response

Author's Rebuttal We thank the reviewers for their detailed review and helpful suggestions, and the editor for their summary. We agree with the raised concerns about the need to clarify the calcium activity in Purkinje cells. We also appreciate knowing what parts were interesting and what seemed less novel. Based on these comments, we've updated the manuscript.

Although not mandated, we decided to move the modeling figure to a supplementary section and downplay its description in the results. As a result, we've cut down on how much we talk about this in the abstract and discussion, and instead, we've given a bit more attention to the findings on how granule cells show patterns of activity over time.

In the newly submitted version of the manuscript, we highlighted in yellow the text that was added and in red, strikethrough the portions that were removed.

In the text below, red indicates reviewers' comments, and green our replies. The line counts in the replies refer to the new version of the manuscript.

Major concerns:

1. The common calcium response in Purkinje cells to simple spike bursts and complex spikes requires more prominence in the interpretation and direct treatment in the Discussion. Complex spikes and simple spikes are almost universally considered independently, and the authors have not presented readers a heuristic for relating the present findings to the literature. At minimum, regarding the claim that PC calcium dynamics can be reconstructed from parallel fiber and climbing fiber inputs, the authors should remind readers in the Discussion that the analysis accounts for the convergence of both input streams on calcium accumulation but is agnostic to the timing of simple and complex spikes. An important comparator will be the paper by Markanday et al Nat Comm 2023. We were not able to find the cited related papers (line 575) as they are omitted from the references list. In this context and considering single innervation, attempting to reconstruct activity of an individual Purkinje cell from a population of inferior olivary neurons is flawed at its premise, and the plausibility and importance of using the entire IO population (versus, per se, a delta function of weights w^IO) would help ground this result in the literature. Relatedly, calling PC responses "activity" in the abstract implies "spiking activity," but one key assumption for relating calcium dynamics to spiking is the presumed direct correlation between intracellular calcium concentration and spike rate. Given the impossibility of disambiguating simple spike bursts and climbing fiber responses, it would be appropriate to assure readers do not interpret these dynamics as spiking activity and therefore to call PC dynamics here "calcium dynamics" or similar.

That said, it is well known at this point that Purkinje cell responses are determined largely by learning and therefore by the relative strengths of the granule cell (and perhaps also inhibitory) synapses. Purkinje cell responses can be made to change dramatically by learning, where the inputs to the neuron do not change, but the Purkinje response changes because of synaptic changes. Given this, we really think there is little value conceptually speaking to this otherwise nicely done analysis. [Ed. note: it's not necessary to remove this analysis, but I recommend considering its framing and presentation relative to these critiques].

We thank the reviewers for the observations. As mentioned, we have downsized the relevance of the modelling part, and we tried to rephrase ambiguous sections to make sure that the reader does not misinterpret the calcium dynamics as simply complex or simple firing rate. Below, specific points are addressed: > "An important comparator will be the paper by Markanday et al Nat Comm 2023. We were not able to find the cited related papers (line 575) as they are omitted from the references list." We now fixed the references to cited papers in line 612, and added the new reference. > "attempting to reconstruct activity of an individual Purkinje cell from a population of inferior olivary neurons is flawed at its premise, and the plausibility and importance of using the entire IO population (versus, per se, a delta function of weights w^IO) would help ground this result in the literature" We do not necessarily want to commit to a delta function of weights, as we know that at this stage of development such a requirement is not so strict, and we have now added a comment on this point in the text as per a later point suggestion (lines 63-64). But, as mentioned, we have overall significantly downsized the prominence to the modeling part. > "Relatedly, calling PC responses "activity" in the abstract implies "spiking activity," but one key assumption for relating calcium dynamics to spiking is the presumed direct correlation between intracellular calcium concentration and spike rate. Given the impossibility of disambiguating simple spike bursts and climbing fiber responses, it would be appropriate to assure readers do not interpret these dynamics as spiking activity and therefore to call PC dynamics here "calcium dynamics" or similar." We agree with this concern, and we have tried to stick to "calcium activity" and "calcium dynamics" (eg: abstract line 17, discussion line 613). We note that we do have a section of the discussion that addresses the issue (lines 625-628).

2. The claim in the abstract that PC activity can be reconstructed from activity of input cells is misleading because the populations were not recorded in parallel or necessarily in the same animals. There is a discussion of the implications under "decoding" section in the methods (line 200), but this caveat and implications should be included in the Discussion. The distinction is complicated by the statement of "parallel characterization" in line 589; while the responses were parallel in the sense that they were all to the same stimulus array, it would be easy to interpret this claim to mean parallel in time. The sequential nature of the recordings does not diminish the findings here but the authors should acknowledge this feature of the data collection outside of the Methods.

We agree with the reviewer, and rephrase the text accordingly. We changed the wording in Discussion, lines 630. We believe that the focus shift from the modeling of the responses to the temporal patterning of GC activity makes further changes in the discussion unnecessary.

4. Presentation of the temporal properties would be improved by avoiding the use of smoothing functions. Those smoothed traces give the appearance of gradual increases in activity, even for granule cells that fire at the very onsets of the visual stimuli. Because the consistency of the responses seems strong, presenting these as unsmoothed peri-stimulus time histograms would give a more accurate portrayal of the responding.

We thank the reviewers for the suggestion. We removed the smoothing in the traces shown in the main figure. Furthermore, we included a new supplementary panel presenting more examples or raw data from individual cells, hoping that it could highlight better the relevance of the observation.

Minor concerns:

1. To support the important claim that there was "no consistent anatomical organization in the localization" of granule cell responses (line 308), it would be useful to see granule cell responses organized by subregion in the extended data.

We decided to take out the statement as considering the reviewer comment we did not consider our "proof of absence" of organization to be strong, and we did not want to expand on this aspect of the observations.

2. It would be helpful for interpreting temporal differences between cell type responses (e.g. line 450) if the authors included specifics on calcium indicator kinetics for the two indicators used. Relatedly, it would be relevant to discuss the lack of sustained responses in PCs in light of their bistability (Sengupta and Thirumalai, 2015).

We added a discussion for this point at line 450: "This is surprising, as the known biophysical properties of PCs would allow them to produce bistable activation (ref: https://doi.org/10.1038/nn0406-459 https://doi.org/10.7554/eLife.09158); it is possible that while stimulus transition could trigger an up-state in some PCs, the transition to down-state could happen before the end of the stimulus".

We also add a sentence for the specifics of calcium indicator kinetics regarding the comparison: "While the indicator expressed in PC was the slower GCaMP6s, we believe that the difference in the indicator time constant (1-1.5 s vs 0.7-1 s) could not account for the different responses to stimuli lasting several seconds." 3. The final sentence of the abstract regarding temporal patterning distorts the novelty of the finding simply because the population in the electrosensory lobe in Kennedy et al are also GCs. The authors should find other wording to highlight the significance of discovering a temporal basis set in the cerebellum proper. Similarly, the description of the Kennedy paper as showing "hints of temporal coding" in granule cells is needlessly vague - theirs was strong evidence for temporal coding in a related but distinct population of cells.

In the abstract we do specify that this is the first observation of such patterning "over many seconds", while in Kennedy et al the maximum observed timescale is patterning over 200 ms; therefore, we stand by the phrasing of our abstract. We did clarify this better in the text, rephrasing the text in this way (line 588):

Although some work has shown hints of temporal coding in GCs (Kennedy et al., 2014) this has never been investigated at the whole GC population scale. -> Previous works have reported the existence of temporal patterning over hundreds of milliseconds in the GCs of the electrosensory lobe, a cerebellum-like structure in the brainstem of mormyrid fish (Kennedy et al., 2014). However, this has never been investigated at the whole GC population, at the timescale of several seconds 4. The point in line 33 that "efficient sensory representations" are required for motor responses seems overstated and should be supported with a citation.

We removed the qualifier "efficient" to avoid overstating.

5. Line 40 reads as though granule cells are situated in the molecular layer We resolved the ambiguity by rephrasing the text.

6. Given the developing circuit under consideration in the study and importance of IO-PC connectivity for the interpretation of the PC integration analysis, it is worth mentioning the exception of multi-innervation during development on line 62.

We added a mention to multi-innervation and some related references in lines 62-63 7. The claim that zebrafish cerebellar neurons are mature by 5 dpf (line 72) is confusing as they are still growing. Perhaps the authors mean "differentiated"? Yes, changed the text to "differentiated".

8. The claim of "no evidence for... oscillatory activity" deserves a caveat that identifying oscillations above 2Hz are precluded by the sampling rate.

We modified the text to identify this caveat, adding "(in the frequency range observable given the sampling rate, i.e. below 2 Hz). " (lines 597-598) 9. Figure 2 title references PCs but they are not included here.

We removed the reference to PCs.

Line 145 "where" typo Line 146 "and" typo Line 373 typo "corresponded mapped" Line 26 "ION" typo We fixed the typos.

## References

[B1] Ahrens MB, Li JM, Orger MB, Robson DN, Schier AF, Engert F, Portugues R (2012) Brain-wide neuronal dynamics during motor adaptation in zebrafish. Nature 485:471–477. 10.1038/nature11057 22622571 PMC3618960

[B2] Albus JS (1971) A theory of cerebellar function. Math Biosci 10:25–61. 10.1016/0025-5564(71)90051-4

[B3] Arenz A, Bracey EF, Margrie TW (2009) Sensory representations in cerebellar granule cells. Curr Opin Neurobiol 19:445–451. 10.1016/j.conb.2009.07.003 19651506

[B4] Bae Y-K, Kani S, Shimizu T, Tanabe K, Nojima H, Kimura Y, Higashijima S-I, Hibi M (2009) Anatomy of zebrafish cerebellum and screen for mutations affecting its development. Dev Biol 330:406–426. 10.1016/j.ydbio.2009.04.013 19371731

[B5] Barker AJ, Helmbrecht TO, Grob AA, Baier H (2017) Detection of whole-field luminance changes by superficial interneurons in the zebrafish tectum. bioRxiv:178970.

[B6] Bosman LW, Konnerth A (2009) Activity-dependent plasticity of developing climbing fiber-Purkinje cell synapses. Neuroscience 162:612–623. 10.1016/j.neuroscience.2009.01.032 19302832

[B7] Bratby P, Sneyd J, Montgomery J (2017) Sequential pattern formation in the cerebellar granular layer. Cerebellum 16:438–449. 10.1007/s12311-016-0820-y 27543118

[B8] Bullock D, Fiala JC, Grossberg S (1994) A neural model of timed response learning in the cerebellum. Neural Netw 7:1101–1114. 10.1016/S0893-6080(05)80161-3

[B9] Buonomano DV, Mauk MD (1994) Neural network model of the cerebellum: temporal discrimination and the timing of motor responses. Neural Comput 6:38–55. 10.1162/neco.1994.6.1.38

[B10] Chen S, Augustine GJ, Chadderton P (2017) Serial processing of kinematic signals by cerebellar circuitry during voluntary whisking. Nat Commun 8:232. 10.1038/s41467-017-00312-1 28794450 PMC5550418

[B11] Crepel F (1982) Regression of functional synapses in the immature mammalian cerebellum. Trends Neurosci 5:266–269. 10.1016/0166-2236(82)90168-0

[B12] D'Angelo E, Mazzarello P, Prestori F, Mapelli J, Solinas S, Lombardo P, Cesana E, Gandolfi D, Congi L (2011) The cerebellar network: from structure to function and dynamics. Brain Res Rev 66:5–15. 10.1016/j.brainresrev.2010.10.002 20950649

[B13] Dean P, Porrill J, Ekerot CF, Jörntell H (2010) The cerebellar microcircuit as an adaptive filter: experimental and computational evidence. Nat Rev Neurosci 11:30–43. 10.1038/nrn2756 19997115

[B14] Eccles JC, Ito M, Szentágothai J (1967) *The cerebellum as a neuronal machine*. Springer Science and Business Media LLC, New York. 10.1007/978-3-662-13147-3

[B15] Eccles JC, Llinás R, Sasaki K (1966) The excitatory synaptic action of climbing fibres on the Purkinje cells of the cerebellum. J Physiol 182:268–296. 10.1113/jphysiol.1966.sp007824 5944665 PMC1357472

[B16] Felix R, Markov DA, Renninger SL, Tomás R, Laborde A, Carey MR, Orger MB, Portugues R (2021) Structural and functional organization of visual responses in the inferior olive of larval zebrafish. bioRxiv:2021.11.29.470378. 10.1523/JNEUROSCI.2352-21.2023 PMC10883660 38195508

[B17] Giovannucci A, et al. (2017) Cerebellar granule cells acquire a widespread predictive feedback signal during motor learning. Nat Neurosci 20:727–734. 10.1038/nn.4531 28319608 PMC5704905

[B18] Gluck MA, Reifsnider ES, Thompson RF (1990) Adaptive signal processing and the cerebellum: models of classical conditioning and VOR adaptation. In: *Neuroscience and connectionist theory*. (Gluck MA, Rumelhart DE, eds), pp 131–185. Hillsdale, NJ: Lawrence Erlbaum Associates, Inc.

[B19] Hamling KR, Tobias ZJC (2015) Mapping the development of cerebellar Purkinje cells in zebrafish. Dev Neurobiol 75:1174–1188. 10.1002/dneu.22275 25655100

[B20] Harmon TC, McLean DL, Raman IM (2020) Integration of swimming-related synaptic excitation and inhibition by olig2+ eurydendroid neurons in larval zebrafish cerebellum. J Neurosci 40:3063–3074. 10.1523/JNEUROSCI.2322-19.2020 32139583 PMC7141884

[B21] Hibi M, Shimizu T (2012) Development of the cerebellum and cerebellar neural circuits. Dev Neurobiol 72:282–301. 10.1002/dneu.20875 21309081

[B22] Hsieh JY, Ulrich B, Issa FA, Wan J, Papazian DM (2014) Rapid development of Purkinje cell excitability, functional cerebellar circuit, and afferent sensory input to cerebellum in zebrafish. Front Neural Circuits 8:147. 10.3389/fncir.2014.00147 25565973 PMC4271617

[B23] Ito M (2000) Mechanisms of motor learning in the cerebellum. Brain Res 886:237–245. 10.1016/S0006-8993(00)03142-5 11119699

[B24] Ito M (2006) Cerebellar circuitry as a neuronal machine. Prog Neurobiol 78:272–303. 10.1016/j.pneurobio.2006.02.006 16759785

[B25] Ito M (2013) Error detection and representation in the olivo-cerebellar system. Front Neural Circuits 7:1. 10.3389/fncir.2013.00001 23440175 PMC3579189

[B26] Johansson F, Jirenhed DA, Rasmussen A, Zucca R, Hesslow G (2014) Memory trace and timing mechanism localized to cerebellar Purkinje cells. Proc Natl Acad Sci U S A 111:14930–14934. 10.1073/pnas.1415371111 25267641 PMC4205653

[B27] Kawato M, Gomi H (1992) The cerebellum and VOR/OKR learning models. Trends Neurosci 15:445–453. 10.1016/0166-2236(92)90008-V 1281352

[B28] Kennedy A, Wayne G, Kaifosh P, Alviña K, Abbott LF, Sawtell NB (2014) A temporal basis for predicting the sensory consequences of motor commands in an electric fish. Nat Neurosci 17:416–422. 10.1038/nn.3650 24531306 PMC4070001

[B29] Knogler LD, Kist AM, Portugues R (2019) Motor context dominates output from Purkinje cell functional regions during reflexive visuomotor behaviours. Elife 8:e42138. 10.7554/eLife.42138 30681408 PMC6374073

[B30] Knogler LD, Markov DA, Dragomir EI, Štih V, Portugues R (2017) Sensorimotor representations in cerebellar granule cells in larval zebrafish are dense, spatially organized, and non-temporally patterned. Curr Biol 27:1288–1302. 10.1016/j.cub.2017.03.029 28434864

[B31] Lister JA, Robertson CP, Lepage T, Johnson SL, Raible DW (1999) Nacre encodes a zebrafish microphthalmia-related protein that regulates neural-crest-derived pigment cell fate. Development 126:3757–3767. 10.1242/dev.126.17.3757 10433906

[B32] Markanday A, Hong S, Inoue J, De Schutter E, Thier P (2023) Multidimensional cerebellar computations for flexible kinematic control of movements. Nat Commun 14:2548. 10.1038/s41467-023-37981-0 37137897 PMC10156706

[B33] Markov DA, Petrucco L, Kist AM, Portugues R (2021) A cerebellar internal model calibrates a feedback controller involved in sensorimotor control. Nat Commun 12:6694. 10.1038/s41467-021-26988-0 34795244 PMC8602262

[B34] Marr D (1969) A theory of cerebellar cortex. J Physiol 202:437–470. 10.1113/jphysiol.1969.sp008820 5784296 PMC1351491

[B35] Medina JF, Garcia KS, Nores WL, Taylor NM, Mauk MD (2000) Timing mechanisms in the cerebellum: testing predictions of a large-scale computer simulation. J Neurosci 20:5516–5525. 10.1523/JNEUROSCI.20-14-05516.2000 10884335 PMC6772322

[B36] Medina JF, Mauk MD (2000) Computer simulation of cerebellar information processing. Nat Neurosci 3:1205–1211. 10.1038/81486 11127839

[B37] Moore JW, Desmond JE, Berthier NE (1989) Adaptively timed conditioned responses and the cerebellum: a neural network approach. Biol Cybern 62:17–28. 10.1007/BF00217657 2590676

[B38] Moreno-Bote R, Beck J, Kanitscheider I, Pitkow X, Latham P, Pouget A (2014) Information-limiting correlations. Nat Neurosci 17:1410–1417. 10.1038/nn.3807 25195105 PMC4486057

[B39] Ohyama T, Nores WL, Murphy M, Mauk MD (2003) What the cerebellum computes. Trends Neurosci 26:222–227. 10.1016/S0166-2236(03)00054-7 12689774

[B40] Pedregosa F, Varoquaux G, Gramfort A (2011) Scikit-learn: machine learning in Python. J Mach Learn Res 12:2825–2830.

[B41] Portugues R, Feierstein CE, Engert F, Orger MB (2014) Whole-brain activity maps reveal stereotyped, distributed networks for visuomotor behavior. Neuron 81:1328–1343. 10.1016/j.neuron.2014.01.019 24656252 PMC4448760

[B42] Sengupta M, Thirumalai V (2015) AMPA receptor mediated synaptic excitation drives state-dependent bursting in Purkinje neurons of zebrafish larvae. Elife 4:e09158. 10.7554/eLife.09158 26416140 PMC4584246

[B43] Štih V, Petrucco L, Kist AM, Portugues R (2019) Stytra: an open-source, integrated system for stimulation, tracking and closed-loop behavioral experiments. PLoS Comput Biol 15:e1006699. 10.1371/journal.pcbi.1006699 30958870 PMC6472806

[B44] Takeuchi M, et al. (2015) Establishment of Gal4 transgenic zebrafish lines for analysis of development of cerebellar neural circuitry. Dev Biol 397:1–17. 10.1016/j.ydbio.2014.09.030 25300581

[B45] Tanaka H, Ishikawa T, Kakei S (2019) Neural evidence of the cerebellum as a state predictor. Cerebellum 18:349–371. 10.1007/s12311-018-0996-4 30627965 PMC6517560

[B46] Wagner MJ, Kim TH, Savall J, Schnitzer MJ, Luo L (2017) Cerebellar granule cells encode the expectation of reward. Nature 544:96–100. 10.1038/nature21726 28321129 PMC5532014

[B47] Williams RW, Herrup K (1988) The control of neuron number. Annu Rev Neurosci 11:423–453. 10.1146/annurev.ne.11.030188.002231 3284447

[B48] Yamazaki T, Tanaka S (2009) Computational models of timing mechanisms in the cerebellar granular layer. Cerebellum 8:423–432. 10.1007/s12311-009-0115-7 19495900 PMC2788136

