# Supplementary figures and images for: Comparing the Representation of a Simple Visual Stimulus across the Cerebellar Network
Source: eNeuro. 2024 Jul 16;11(7):ENEURO.0023-24.2024. doi: 10.1523/ENEURO.0023-24.2024 (PMC11255392; doi:10.1523/ENEURO.0023-24.2024)

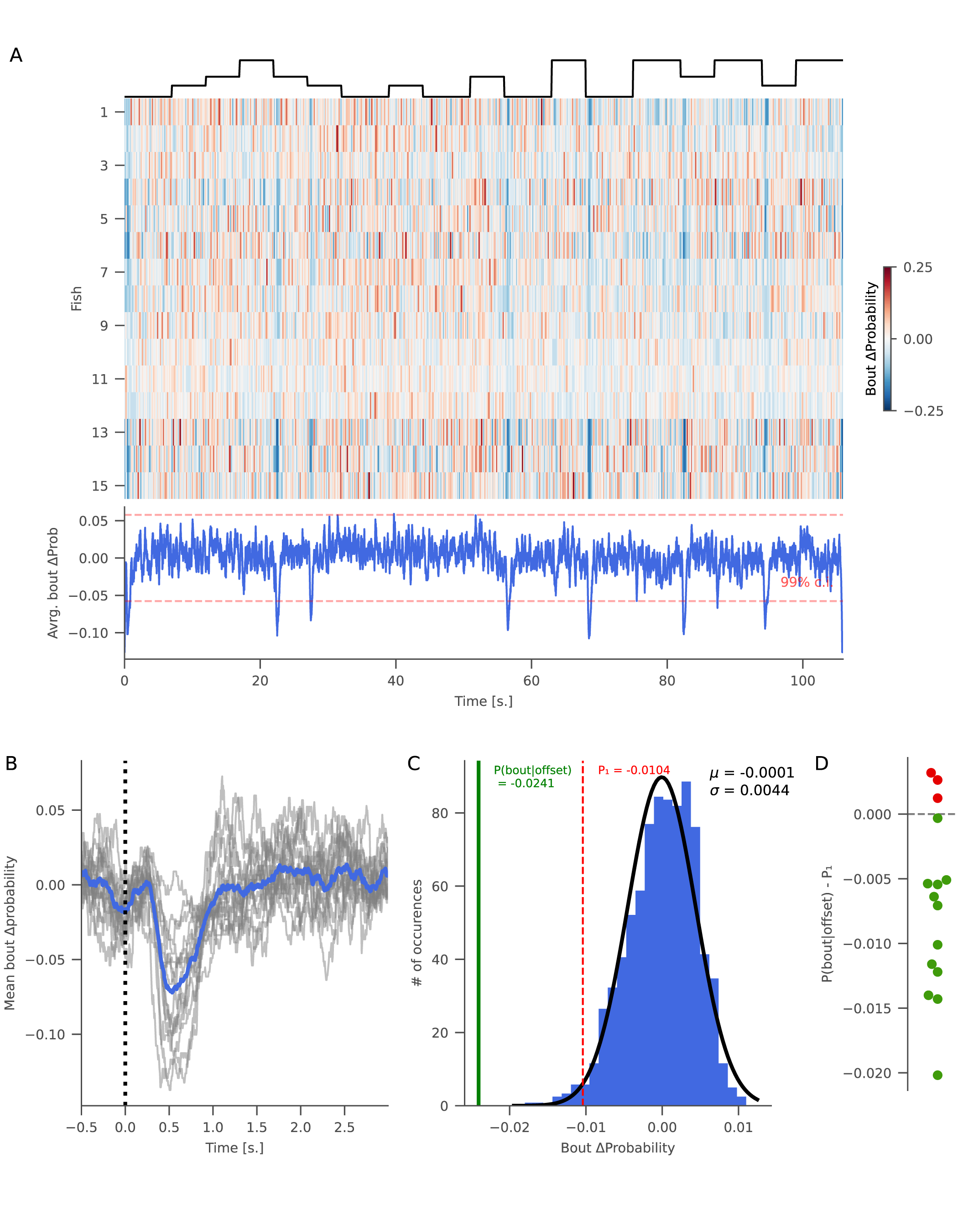

Supplement: Figure 1-1 — A) Average normalized bout probability for each individual fish across all stimulus presentations (heatmap), and average probability across all fish (blue line). B) Bout probability changes on a 3.5 s window around luminance offsets. Average bout probability across all off transitions is shown for each fish in gray lines, and the average bout probability across all fish is plotted in blue. C) The average bout probability at offset (during the 1.5 s following luminance changes, green line) across all fish, compared to the 1st percentile (red line) of a dataset generated via bootstrapping. D) Difference between the bout probability at offset and the 1st percentile for the bootstrapping analysis performed individually on each fish. Fish labeled in green correspond to animals where the bout probability was smaller than the 1st percentile. Download Figure 1-1, TIF file. [file eneuro-11-ENEURO.0023-24.2024-s001.tif]

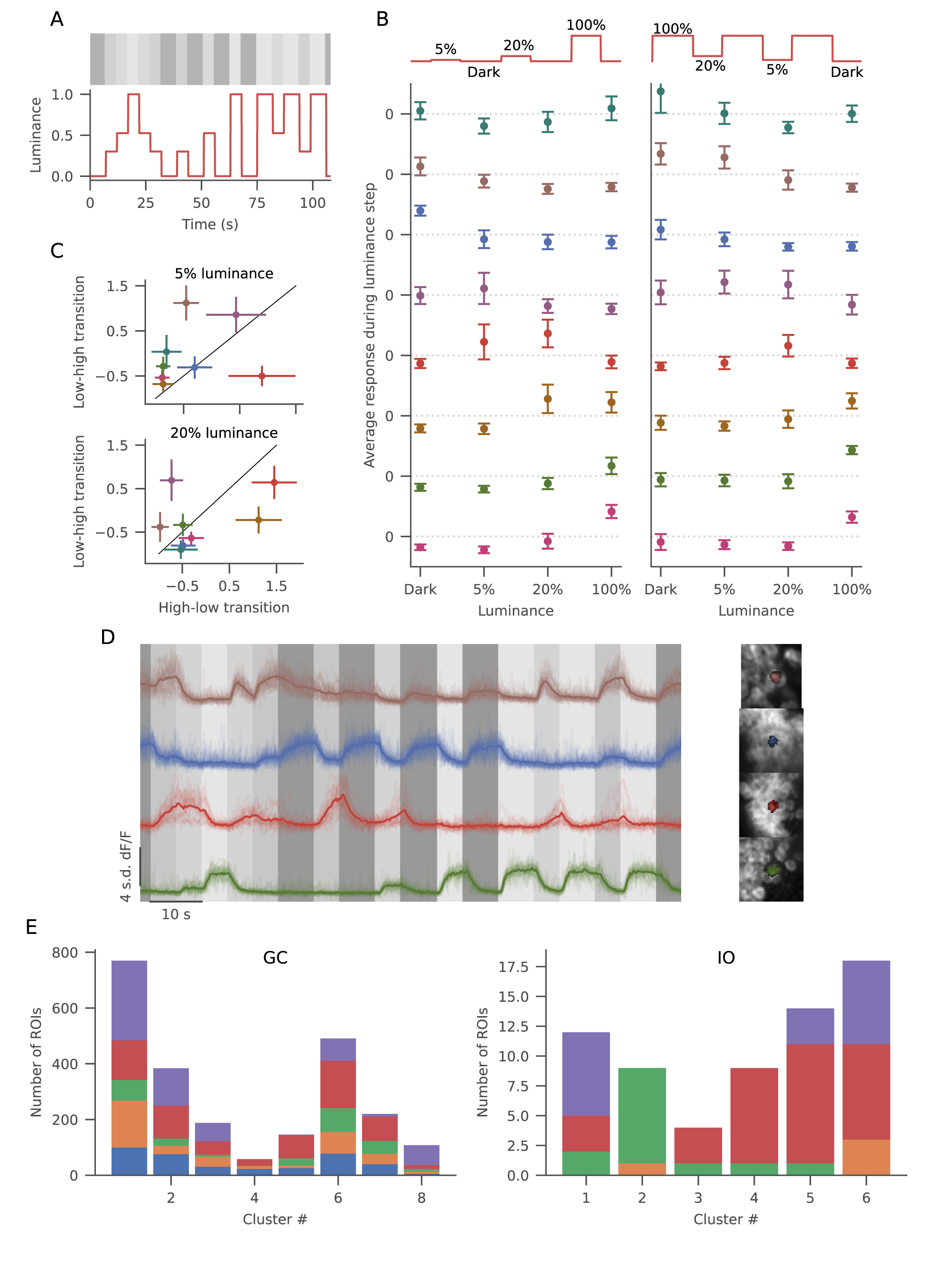

Supplement: Figure 2-1 — A) Schema of the stimulus presented, with the color scheme used for all figures mapped on top of its luminance profile. B) Average fluorescence during the upward luminance steps from minimum luminance (left) and during downward luminance steps from maximum luminance (right) for each GC cluster. C) History dependence of luminance responses for all GC clusters. Average normalized fluorescence during the presentation of the same two intermediate levels of luminance (low intermediate: above, high intermediate: below), compared in epochs when it was reached from a higher (x values) or lower (y values) luminance level. Clusters that deviate from the diagonal are the ones showing the strongest temporal history dependence (color-coded according to Figure 2A). D) Example of GC responses from various clusters, with individual stimulus repetitions (thin lines), average (thick line), and the ROI morphology (on the right). E) Contribution of individual fish to the observed clusters for GCs and IONs. Each color corresponds to one fish. Download Figure 2-1, TIF file. [file eneuro-11-ENEURO.0023-24.2024-s002.tif]

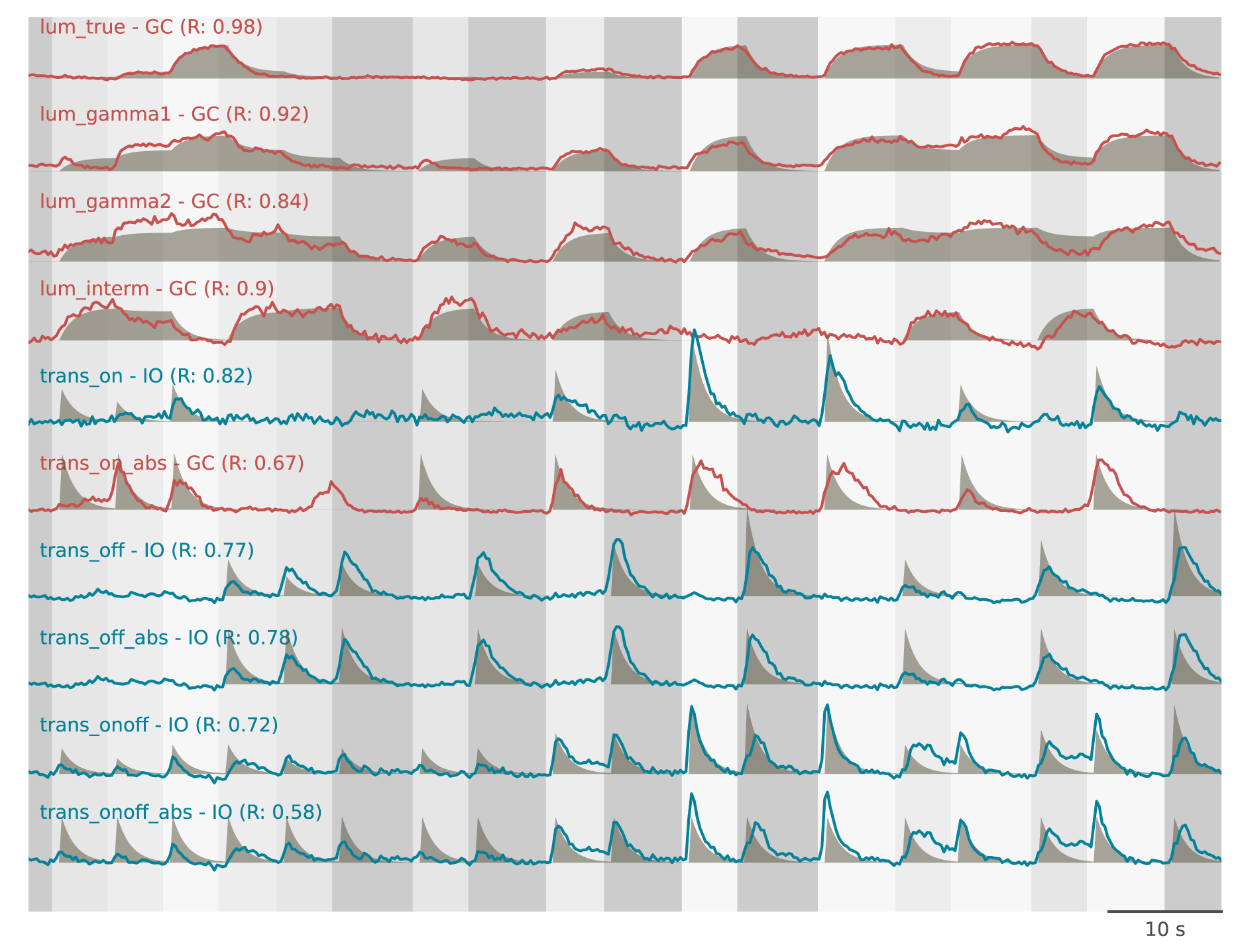

Supplement: Figure 3-1 — All regressors used in the regression analysis (shades), and the best scoring ROI for each regressor (lines). While for luminance-related regressors the highest correlation values were always from GCs, for transition-related regressors most of the best scoring ROIs were from IONs. Download Figure 3-1, TIF file. [file eneuro-11-ENEURO.0023-24.2024-s003.tif]

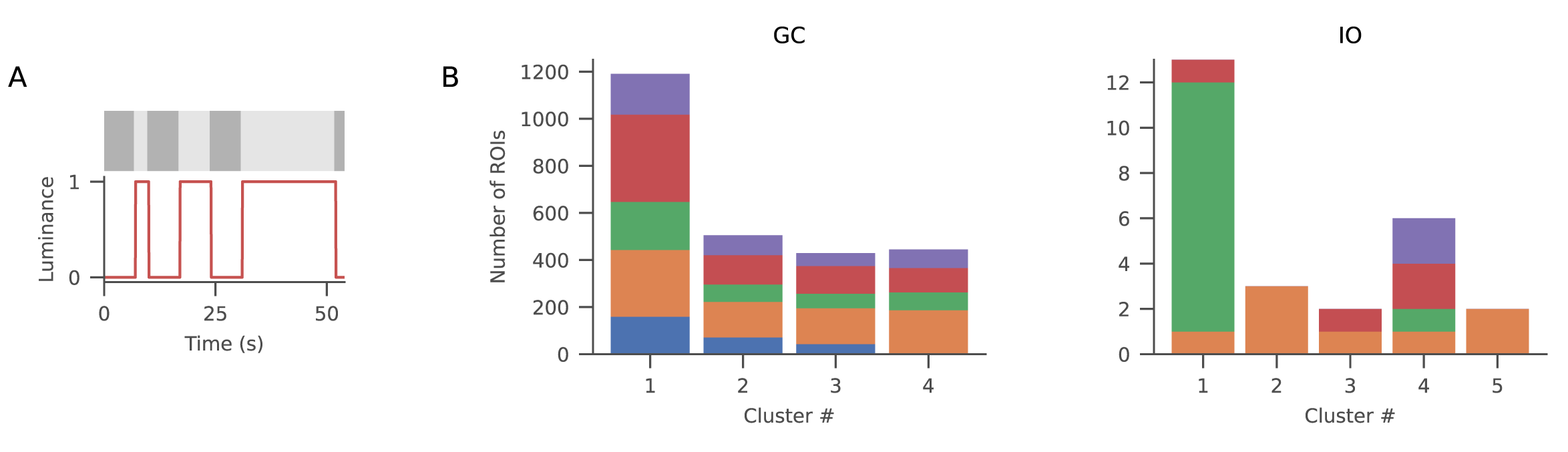

Supplement: Figure 4-1 — A) Schema of the stimulus presented, with the color scheme used for all figures mapped on top of its luminance profile. E) Contribution of individual fish to the observed clusters for GCs and IONs. Each color corresponds to one fish. Download Figure 4-1, TIF file. [file eneuro-11-ENEURO.0023-24.2024-s004.tif]

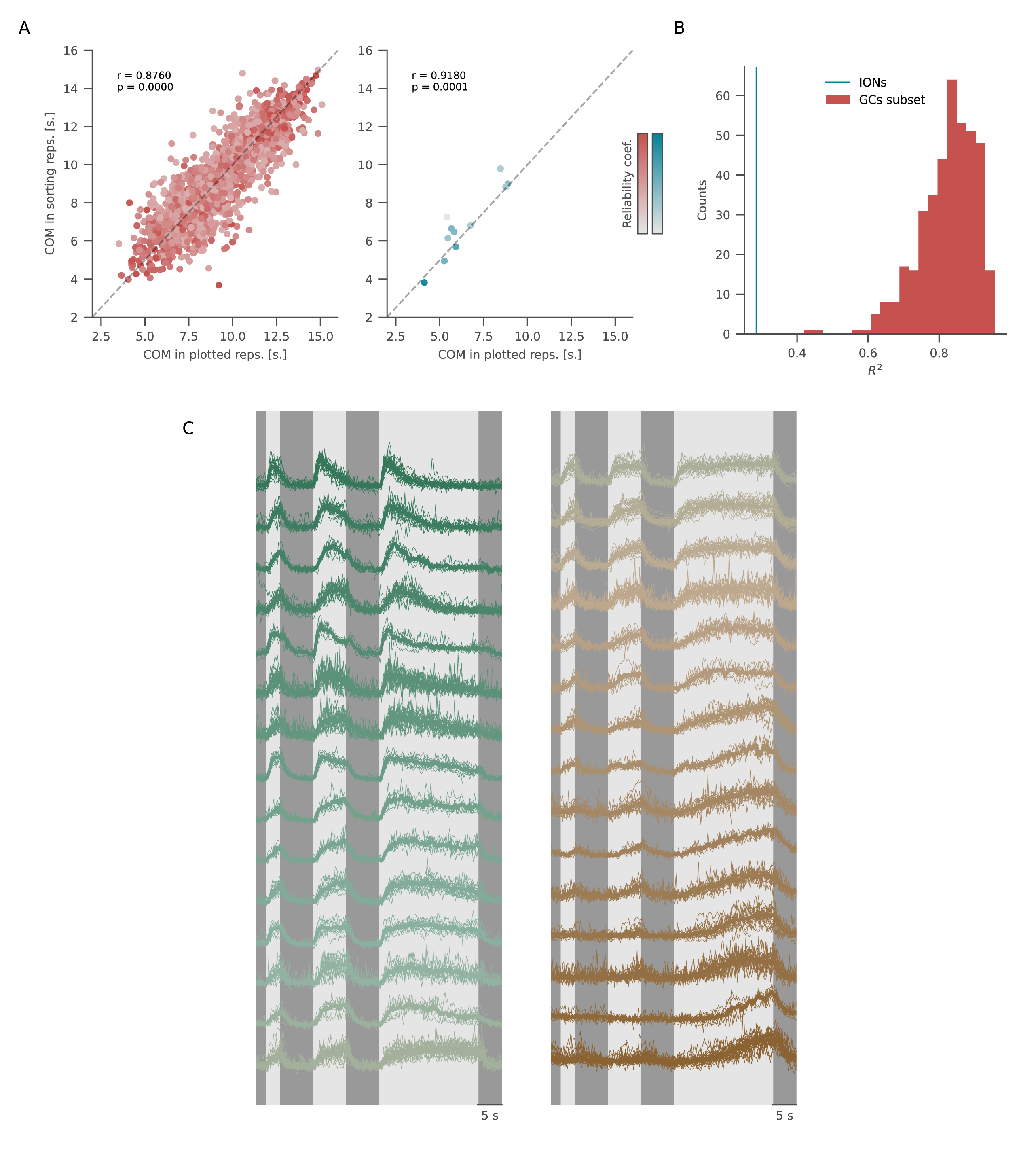

Supplement: Figure 5-1 — A) In order to cross-validate our sorting of ROI responses, the COM for each neuron was calculated based on the average response during half of its repetitions, and the other half of the repetitions were used to plot the figures shown in Figure 5. Figure 5-1A shows, for each ROI, the COM calculated on each half of the repetition. B) Histogram of the R2 values between predicted and actual time from stimulus onset, as decoded from 200 subsamples of GCs. The blue line marks the R2 value obtained using the same number of IONs. C) Additional examples for single GC traces exhibiting temporally-patterned responses, from early- to late-responding neurons (color coded from green to brown). Average response (thick lines) is superimposed to single repetitions of stimulus presentation (thin lines). Download Figure 5-1, TIF file. [file eneuro-11-ENEURO.0023-24.2024-s005.tif]

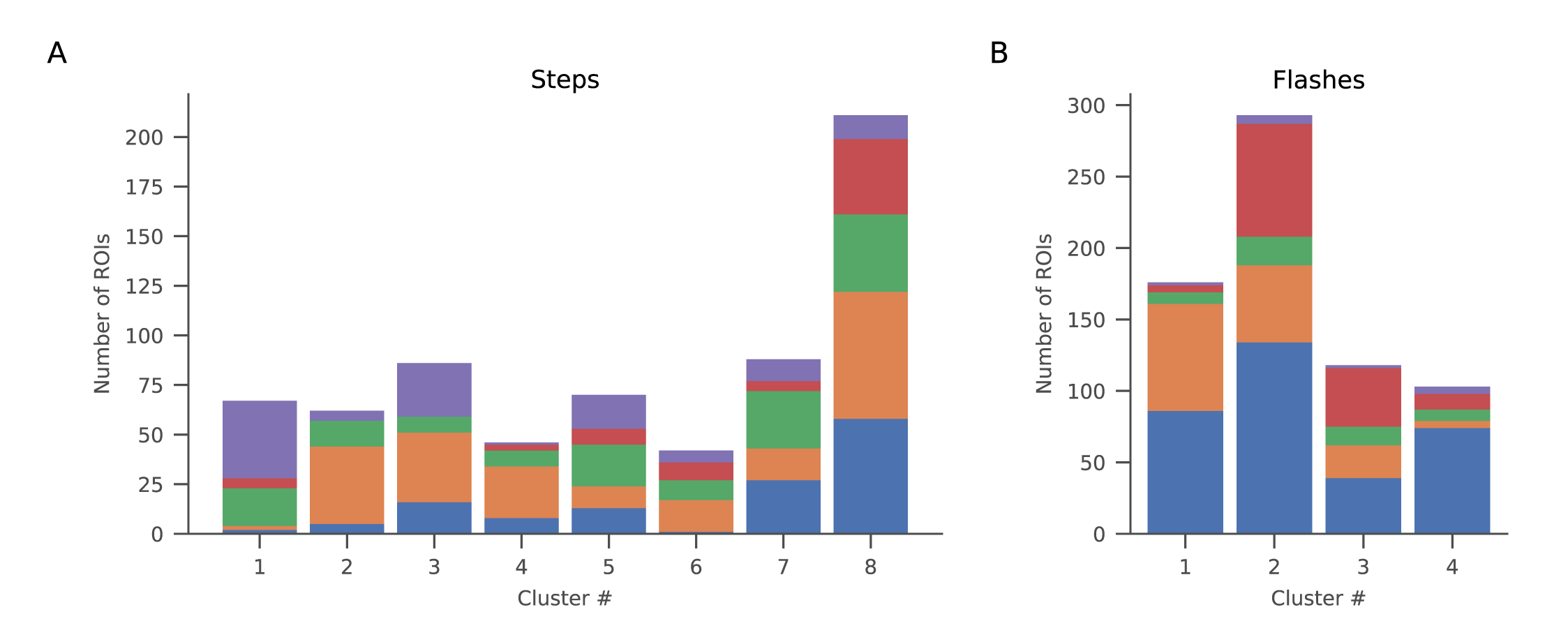

Supplement: Figure 6-1 — A) Contribution of individual fish to the observed clusters for PCs in the steps protocol. Each color corresponds to one fish. B) Contribution of individual fish to the observed clusters for PCs in the flashes protocol. Download Figure 6-1, TIF file. [file eneuro-11-ENEURO.0023-24.2024-s006.tif]

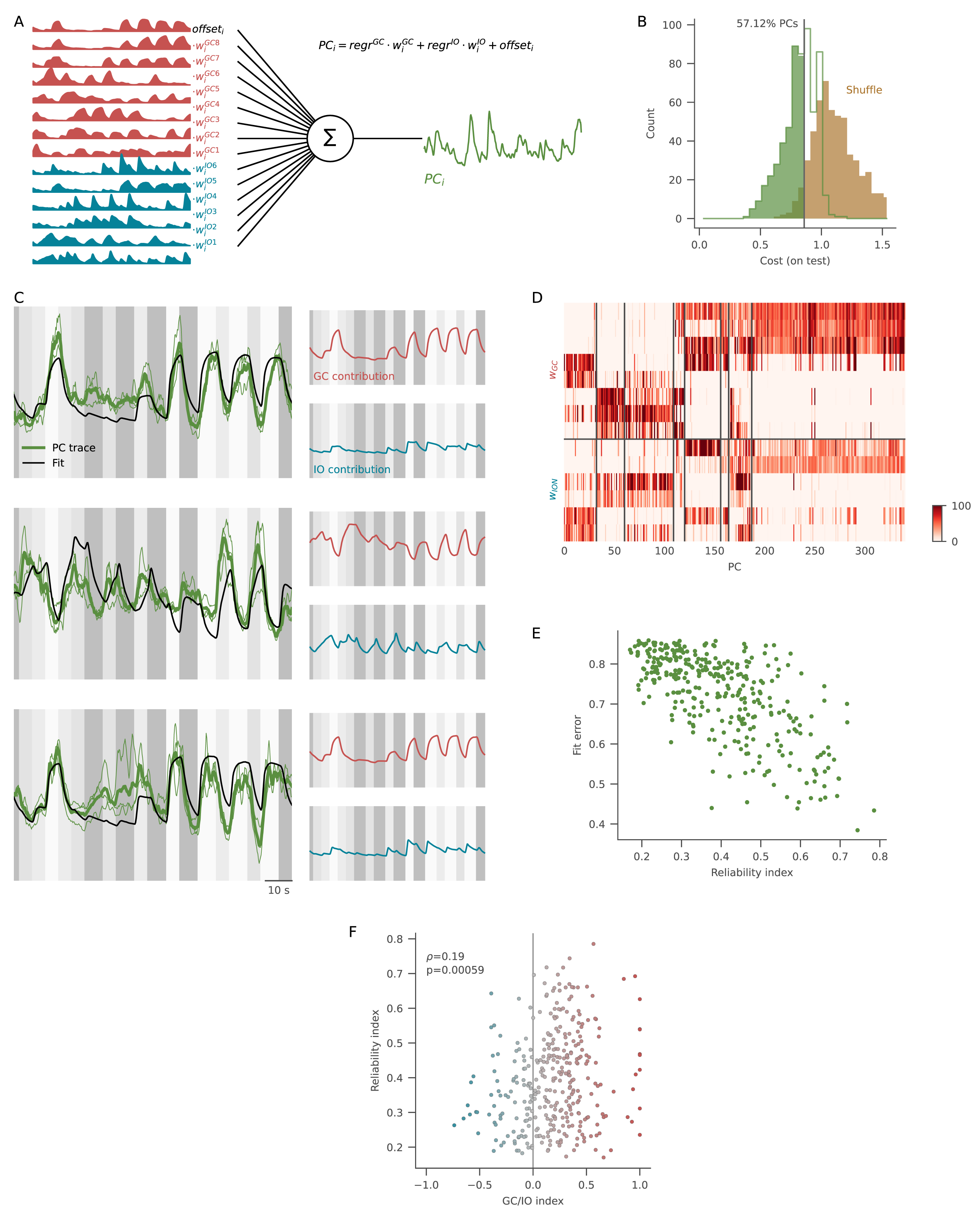

Supplement: Figure 6-2 — A) Schema of the modeling approach. (Left) PCs receive inputs from IONs and GCs. Starting from GC and IO response clusters (center) we linearly combined them trying to reconstruct each PC activity (right). B) Histogram of costs (L2) on the test traces from all PCs (green line), compared to a shuffle distribution (brown shade) that was obtained calculating costs after a random regressor-wise reshuffling of the weight matrix (i.e., all values from each individual regressor were kept and reshuffled in new random combinations). A threshold was defined to ensure that only 5% of these random fits could have a lower cost. The green shade indicates the data that were kept after such selection. C) Examples of individual fits. (Left) Average PC response (thick green line) calculated on the individual test repetitions (thin green lines), and reconstructed trace from the model (black line). (Right) Red, trace reconstructed with GC coefficients only; blue: trace reconstructed with IO coefficients only. D) Matrix of weights assigned to each regressor (rows) for all PC ROIs (columns). Vertical gray lines separate PCs clustered together in Figure 6A. E) Correlation between the reliability index and the fit error, including only PCs for which the fit was considered better-than-random. F) Scatter plot showing the relation between the GC/IO weights ratio vs. cell reliability. Each dot represents the two values for a single PC cell, color coded by the GC/IO index. The response of PCs dominated by GC inputs are more reliable compared to cells dominated by IO inputs (Spearman rho: 0.19, p = 5.8*10-4). Download Figure 6-2, TIF file. [file eneuro-11-ENEURO.0023-24.2024-s007.tif]
